# Psoriasis Patients Are Enriched for Genetic Variants That Protect against HIV-1 Disease

**DOI:** 10.1371/journal.pgen.1002514

**Published:** 2012-02-16

**Authors:** Haoyan Chen, Genki Hayashi, Olivia Y. Lai, Alexander Dilthey, Peter J. Kuebler, Tami V. Wong, Maureen P. Martin, Marcelo A. Fernandez Vina, Gil McVean, Matthias Wabl, Kieron S. Leslie, Toby Maurer, Jeffrey N. Martin, Steven G. Deeks, Mary Carrington, Anne M. Bowcock, Douglas F. Nixon, Wilson Liao

**Affiliations:** 1 Department of Dermatology, University of California San Francisco, San Francisco, California, United States of America; 2 Department of Statistics, University of Oxford, Oxford, United Kingdom; 3 Division of Experimental Medicine, University of California San Francisco, San Francisco, California, United States of America; 4 Cancer and Inflammation Program, Laboratory of Experimental Immunology, National Cancer Institute, Frederick, Maryland, United States of America; 5 Department of Pathology, Stanford University, Palo Alto, California, United States of America; 6 Department of Microbiology and Immunology, University of California San Francisco, San Francisco, California, United States of America; 7 Department of Epidemiology and Biostatistics, University of California San Francisco, San Francisco, California, United States of America; 8 HIV/AIDS Program, Department of Medicine, University of California San Francisco, San Francisco, California, United States of America; 9 Division of Human Genetics, Department of Genetics, Washington University School of Medicine, Saint Louis, Missouri, United States of America; Duke University, United States of America

## Abstract

An important paradigm in evolutionary genetics is that of a delicate balance between genetic variants that favorably boost host control of infection but which may unfavorably increase susceptibility to autoimmune disease. Here, we investigated whether patients with psoriasis, a common immune-mediated disease of the skin, are enriched for genetic variants that limit the ability of HIV-1 virus to replicate after infection. We analyzed the HLA class I and class II alleles of 1,727 Caucasian psoriasis cases and 3,581 controls and found that psoriasis patients are significantly more likely than controls to have gene variants that are protective against HIV-1 disease. This includes several HLA class I alleles associated with HIV-1 control; amino acid residues at HLA-B positions 67, 70, and 97 that mediate HIV-1 peptide binding; and the deletion polymorphism rs67384697 associated with high surface expression of HLA-C. We also found that the compound genotype *KIR3DS1* plus *HLA-B Bw4-80I*, which respectively encode a natural killer cell activating receptor and its putative ligand, significantly increased psoriasis susceptibility. This compound genotype has also been associated with delay of progression to AIDS. Together, our results suggest that genetic variants that contribute to anti-viral immunity may predispose to the development of psoriasis.

## Author Summary

Individuals with autoimmune disease generally demonstrate excessive immune system activation, leading to inflammation and damage of specific target organs. However, in some cases the detrimental effects of an overactive immune system might be counterbalanced by a beneficial effect in protecting against certain infections. In this study, we investigated whether patients with psoriasis, a common autoimmune disease of the skin, harbor genetic variants that are associated with an enhanced ability to limit replication of the HIV-1 virus. We profiled the HLA (human leukocyte antigen) immune genes located on chromosome 6 in 1,727 Caucasian psoriasis cases and 3,581 healthy controls and found that psoriasis patients are significantly more likely than controls to have gene variants that are protective against HIV-1 disease. We found that this enrichment for HIV-1 protective variants was unique to psoriasis and largely absent in patients with other autoimmune or inflammatory diseases such as rheumatoid arthritis, Crohn's disease, type 1 diabetes, type 2 diabetes, and coronary artery disease. Our results suggest the possibility that the excessive skin inflammation in psoriasis may be associated with activation of anti-viral immune pathways that were important to human ancestors who encountered viruses similar to HIV-1.

## Introduction

Psoriasis is an immune-mediated, inflammatory skin disease that is associated with arthritis and other systemic co-morbidities [1]. Psoriasis is a highly heritable condition, with a monozygotic twin concordance rate of 70% [2] and an estimated sibling recurrence risk λ_s_ of 4–11 [3]. Linkage studies [4]–[6] and genome-wide association studies (GWAS) [7]–[11] have identified over 20 psoriasis susceptibilities alleles. However, the locus consistently displaying the strongest association signal, by many orders of magnitude, is the major histocompatibility complex (MHC).

We were intrigued by the observation that several of the most highly significant SNPs from psoriasis GWAS were identical to the top SNPs from GWAS of HIV-1 virologic control, a clinical phenotype whereby certain HIV-1 infected individuals, termed “HIV-1 controllers,” are able to maintain low levels of plasma HIV-1 RNA in the absence of antiretroviral therapy and who generally do not develop clinical symptoms [12]. For example, rs2395029 within the MHC gene *HCP5* and a proxy for HLA-B*57, was identified in a psoriasis GWAS as the SNP with the largest odds ratio, OR = 4.1, p = 2.13×10^−26^ [8]. This same SNP has been shown to be the first or second most significant SNP in three GWAS of HIV-1 control [13]–[15]. The two most significant SNPs identified in multiple psoriasis GWAS are rs10484554 and rs12191877 near HLA-C (r^2^ = 1 with each other in Europeans) [8]–[10]. rs10484554 and rs12191877 were found to be associated with HIV-1 control [Supplementary Materials in [13], [15]] and both are in moderate linkage disequilibrium (r^2^ = 0.33) with rs9264942, another top SNP for HIV-1 control [14]–[16].

The relationship between psoriasis and HIV-1 is also interesting because of the clinical observation that HIV-1 infection can exacerbate existing psoriasis or trigger new-onset psoriasis [17]. As HIV-1 infection progresses and CD4+ T cell counts decrease, psoriasis can worsen [18], [19]. This has puzzled dermatologists and infectious disease clinicians because it has been convincingly established that psoriasis is an immune disorder that is mediated through activation of T cells. Several explanations for this “psoriasis HIV-1 paradox” have been proposed, including HIV-1 induced destruction of regulatory CD4+ T cells [20], an increase in number of memory CD8+ T cells late in disease [21], HIV-1 proteins acting as superantigens [22], or co-stimulation through traditional antigenic presentation [20].

Due to these genetic and clinical observations, we pursued a more in-depth analysis of the HLA region in psoriasis to determine whether patients with psoriasis are enriched for the major genetic determinants of HIV-1 control. The psoriasis data generated in this study were compared to the largest GWAS for HIV-1 control performed to date, involving 516 cases and 1,196 controls and for which detailed HLA allele information was available [15].

## Results

### Accurate Imputation of HLA Alleles

We imputed to four-digit resolution the HLA class I alleles (-A, -B, -C) and HLA class II alleles (-DQA1, -DQB1, -DRB1) of 1,727 psoriasis cases and 3,581 healthy controls which were obtained from 3 separate case-control cohorts of European ancestry (Table S1). Imputation was performed using the software HLA*IMP, which has been shown to have an accuracy of at least 96% for class I loci and 92% for Class II loci [23]. To further validate the accuracy of our imputation, we compared the imputed HLA alleles to empirically obtained HLA class I alleles for a subset of our samples (n = 98). The concordance was 566/581 alleles (97.4%), indicating that the imputation was of high accuracy. A sensitivity analysis examining the imputation accuracy of low frequency HLA alleles (allele frequency between 1% and 5%) demonstrated similar high accuracy (177/179 alleles = 98.9%). Only HLA alleles with a minor allele frequency greater than 1% in the control group were used for subsequent analyses.

### HLA Association Testing Identifies a Similar Genetic Architecture between Psoriasis and HIV-1 Control

We tested all imputed HLA alleles for association with psoriasis using logistic regression, adjusting for gender, ancestry, and cohort. The top ten HLA associations for psoriasis are shown in Table 1 (Full four-digit and two-digit results in Tables S2 and S3, respectively). Overall, we observed a striking pattern in which the HLA alleles which are enriched in psoriasis patients are also enriched in HIV-1 controllers, and the HLA alleles which have decreased frequency in psoriasis patients are also decreased in HIV-1 controllers. We found that psoriasis patients are highly enriched for HLA-B*57:01 (12.5% in cases vs 3.9% in controls, p = 5.50×10^−42^, OR = 3.61), which in multiple studies has been shown to be the most significant predictor of both HIV-1 control and delayed progression time to AIDS [14], [15], [24]–[27]. Psoriasis patients also display a significant enrichment of the HIV-1 control allele B*13:02, whereas they display a relative paucity of B*07:02, B*40:01, and C*04:01 which are associated with lack of virologic control [15]. The HLA allele B*35, almost always seen with C*04:01, and the most significant HLA allele associated with rapid progression to AIDS [28], [29], was significantly protective against psoriasis in our dataset (p = 3.20×10^−6^, OR = 0.65 [0.54–0.78]). HLA-B*35 alleles can be segregated into B*35-Px and B*35-PY alleles, where Px alleles bind peptides with hydrophobic, non-tyrosine residues at position 9 and PY alleles bind peptides with tyrosine at position 9. It has been shown that the influence of HLA-B*35 in accelerating progression to AIDS is mostly attributable to HLA-B*35-Px alleles [30]. In our psoriasis dataset, the B*35-Px alleles B*35:02 and B*35:03 together demonstrated a stronger effect on psoriasis protection (p = 2.9×10^−4^, OR = 0.47 [0.31–0.71]) than the B*35-PY allele B*35:01 (p = 5.86×10^−3^, OR = 0.74 [0.60–0.92]).

**Table 1:** Top ten classical HLA alleles associated with psoriasis, and comparison to HIV-1 controllers as published in [15].

Psoriasis						HIV			
Rank	Allele	Frequency in cases (n = 1727)	Frequency in controls (n = 3581)	P-value	OR	Frequency in controllers (n = 516)	Frequency in non-controllers (n = 1196)	P-value	OR
1	C*06:02	0.253	0.098	2.91E-77	3.57	0.195	0.081	2.10E-19	2.97
2	B*57:01†	0.125	0.039	5.50E-42	3.61	0.139	0.032	1.40E-26	5.48
3	DQA1*02:01	0.256	0.152	3.40E-24	1.99	0.184	0.117	1.10E-06	1.67
4	DQB1*03:03	0.118	0.050	1.18E-22	2.69	0.096	0.045	5.40E-09	2.48
5	DRB1*07:01	0.246	0.149	1.98E-20	1.90	0.183	0.117	1.10E-06	1.68
6	B*13:02†	0.063	0.025	6.20E-17	2.77	0.041	0.019	1.80E-03	2.06
7	A*01:01	0.215	0.165	1.98E-08	1.40	0.139	0.149	NS	1
8	C*04:01†	0.074	0.120	2.21E-08	0.63	0.058	0.109	5.00E-08	0.42
9	B*07:02†	0.098	0.122	3.40E-06	0.71	0.061	0.133	1.40E-07	0.45
10	B*40:01†	0.038	0.052	7.24E-06	0.60	0.029	0.054	8.10E-03	0.55

Alleles marked with † have an independent effect on HIV-1 control. A high degree of similarity is observed between psoriasis and HIV-1 control with respect to the magnitude and direction of the associated alleles. P values and ORs for psoriasis samples were adjusted by ancestry, gender, and cohort. ORs greater than 1.0 correspond to psoriasis susceptibility and control of HIV-1, whereas ORs less than 1.0 correspond to decreased psoriasis susceptibility and lack of virologic control. NS, not significant.

### Stepwise Regression Modeling Identifies HLA Alleles with Independent Effects on Psoriasis Susceptibility, Including the HIV-1 Control Alleles B*57:01 and B*27:05

To identify HLA alleles independently associated with psoriasis, we performed stepwise regression modeling, first conditioning the association results on the top allele HLA-C*06:02, and then adding alleles to the model in a stepwise manner. We identified HLA-C*06:02, B*38:01, A*02:01, B*39:01, B*27:05, B*08:01, B*14:02, B*55:01, and B*57:01 as HLA class I alleles independently associated with psoriasis (Table 2). In the multivariate regression model including all of these alleles, the HIV-1 viral control alleles B*57:01 and B*27:05 both had significant effect on psoriasis susceptibility (OR = 1.52 and 1.75, respectively). The contribution of B*27:05 was more apparent in the regression model than when B*27:05 was analyzed as a single allele (p = 0.016, OR = 1.32 [1.05–1.66]). The HIV-1 progression allele B*35 remained independently associated with psoriasis after conditioning on the top allele C*06:02 (p = 0.0064, OR = 0.77 [0.63–0.93]), but further conditioning on B*38:01 and A*02:01 resulted in a residual association signal for B*35 of p = 0.0168, OR = 0.78 [0.64–0.96]).

**Table 2:** HLA class I alleles identified by stepwise logistic regression as independently associated with psoriasis.

Allele	Frequency in cases	Frequency in controls	Stepwise Univariate		Multivariate	
			P value	OR	P value	OR
HLA-C*06:02	0.253	0.098	2.91E-77	3.57	1.03E-45	3.65
HLA-B*38:01	0.035	0.023	2.69E-07	2.21	9.13E-10	2.68
HLA-A*02:01	0.298	0.254	3.68E-05	1.27	2.22E-06	1.32
HLA-B*39:01	0.022	0.012	1.72E-04	2.09	9.17E-06	2.40
HLA-B*27:05	0.049	0.035	1.45E-04	1.62	1.15E-05	1.75
HLA-B*08:01	0.100	0.109	3.01E-04	1.36	1.88E-05	1.45
HLA-B*14:02	0.031	0.030	4.36E-04	1.73	2.34E-04	1.77
HLA-B*55:01	0.023	0.016	4.87E-04	1.84	3.61E-04	1.86
HLA-B*57:01	0.125	0.039	5.77E-04	1.52	5.77E-04	1.52

We also performed stepwise regression modeling combining class I and class II HLA alleles. At 4 digit resolution, C*06:02, B*38:01, DQB1*05:02, DQB1*06:04, and A*02:01 were found to be independent risk factors; however, at 2 digit resolution, the class II alleles DQB1*05 and DQB1*06 were no longer significant (data not shown).

### The Extended Haplotype B*57:01–C*06:02–DQA1*02:01–DQB1*03:03–DRB1*07:01 Is Associated with Both Psoriasis Susceptibility and HIV-1 Control

We performed haplotype analysis in psoriasis patients and HIV-1 controllers to help understand how combinations of HLA alleles contribute to the observed association signals. We estimated the frequency of HLA haplotypes in our psoriasis case-control cohort as well as in 214 Caucasian HIV-1 infected individuals (52 HIV-1 controllers, 162 non-controllers) in the SCOPE cohort, whose HLA class I and II alleles had been previously genotyped. Our analysis revealed that both psoriasis patients and HIV-1 controllers are highly enriched for the B*57:01–C*06:02 haplotype as well as the extended haplotype B*57:01–C*06:02–DQA1*02:01–DQB1*03:03–DRB1*07:01, thus explaining why these individual alleles are associated with both phenotypes (Table 3 and Table 4). We found that the association of DQA1*02:01, DQB1*03:03, and DRB1*07:01 with psoriasis was nearly completely due to the effects of C*06:02 or B*57:01, since conditioning DQA1*0201 and DRB1*0701 on C*06:02 resulted in p = 0.017, OR = 1.21 and p = 0.199, OR = 1.11, respectively; and conditioning DQB1*0303 on B*57:01 resulted in p = 0.038, OR = 1.33. Thus, the primary genetic determinants of both psoriasis and HIV-1 control reside within the class I alleles.

**Table 3:** Association testing of HLA B–C haplotypes with psoriasis and HIV-1 control.

Psoriasis						HIV			
HLA-B	HLA-C	Frequency in cases (n = 1727)	Frequency in controls (n = 3581)	P-value	OR	Frequency in controllers (n = 52)	Frequency in non-controllers (n = 162)	P-value	OR
57:01	06:02	0.118	0.034	3.06E-65	3.86	0.183	0.046	3.62E-05	4.58
13:02	06:02	0.059	0.024	3.25E-20	2.56	0.029	0.015	NS	-
37:01	06:02	0.025	0.013	3.25E-06	1.99	0.019	0.009	NS	-
38:01	12:03	0.034	0.022	4.60E-04	1.54	-	-	NS	-
18:01	07:01	0.013	0.022	6.25E-04	0.56	-	-	NS	-
44:03	16:01	0.019	0.029	1.00E-03	0.63	0.010	0.031	NS	-
07:02	07:02	0.092	0.112	1.28E-03	0.80	0.087	0.083	NS	-
40:01	03:04	0.035	0.048	2.13E-03	0.72	0.019	0.059	NS	-
27:05	01:02	0.019	0.012	3.20E-03	1.62	0.067	0.025	NS	-
18:01	12:03	0.012	0.019	0.005	0.61	0.010	0.019	NS	-

The B*57:01–C*06:02 haplotype is highly enriched in both psoriasis and HIV-1 control patients. The limited sample size of the HIV cohort precludes the detection of smaller effects. Only haplotypes with p<0.01 are shown.

**Table 4:** Association testing of extended class I and II HLA haplotypes with psoriasis and HIV-1 control.

Psoriasis									HIV			
HLA-B	HLA-C	HLA-DQA1	HLA-DQB1	HLA-DRB1	Frequency in cases	Frequency in controls	P-value	OR	Frequency in cases	Frequency in controls	P-value	OR
57:01	06:02	02:01	03:03	07:01	0.088	0.022	1.51E-34	4.30	0.106	0.034	0.0084	3.35
13:02	06:02	02:01	02:02	07:01	0.042	0.015	3.50E-11	2.96	0.010	0.037	NS	-
40:01	03:04	03:01	03:02	04:04	0.003	0.013	6.23E-06	0.22	0.028	0.031	NS	-
35:01	04:01	01:01	05:01	01:01	0.013	0.026	4.00E-06	0.52	-	-	-	-
40:01	03:04	01:02	06:04	13:02	0.005	0.016	7.60E-04	0.32	-	-	-	-

The B*57:01–C*06:02–DQA1*02:01–DQB1*03:03–DRB1*07:01 haplotype is highly enriched in both psoriasis and HIV-1 control patients. Note: HLA-DQA1 was not genotyped in the HIV cohort, thus the frequencies reflect HLA B –C–DQB1–DRB1 haplotypes. Only haplotypes with p<0.01 are shown.

### Amino Acids within *HLA-B* That Are Predictive of HIV-1 Viral Load Are Concordant between HIV-1 Controllers and Psoriasis Patients

**Figure 1:**
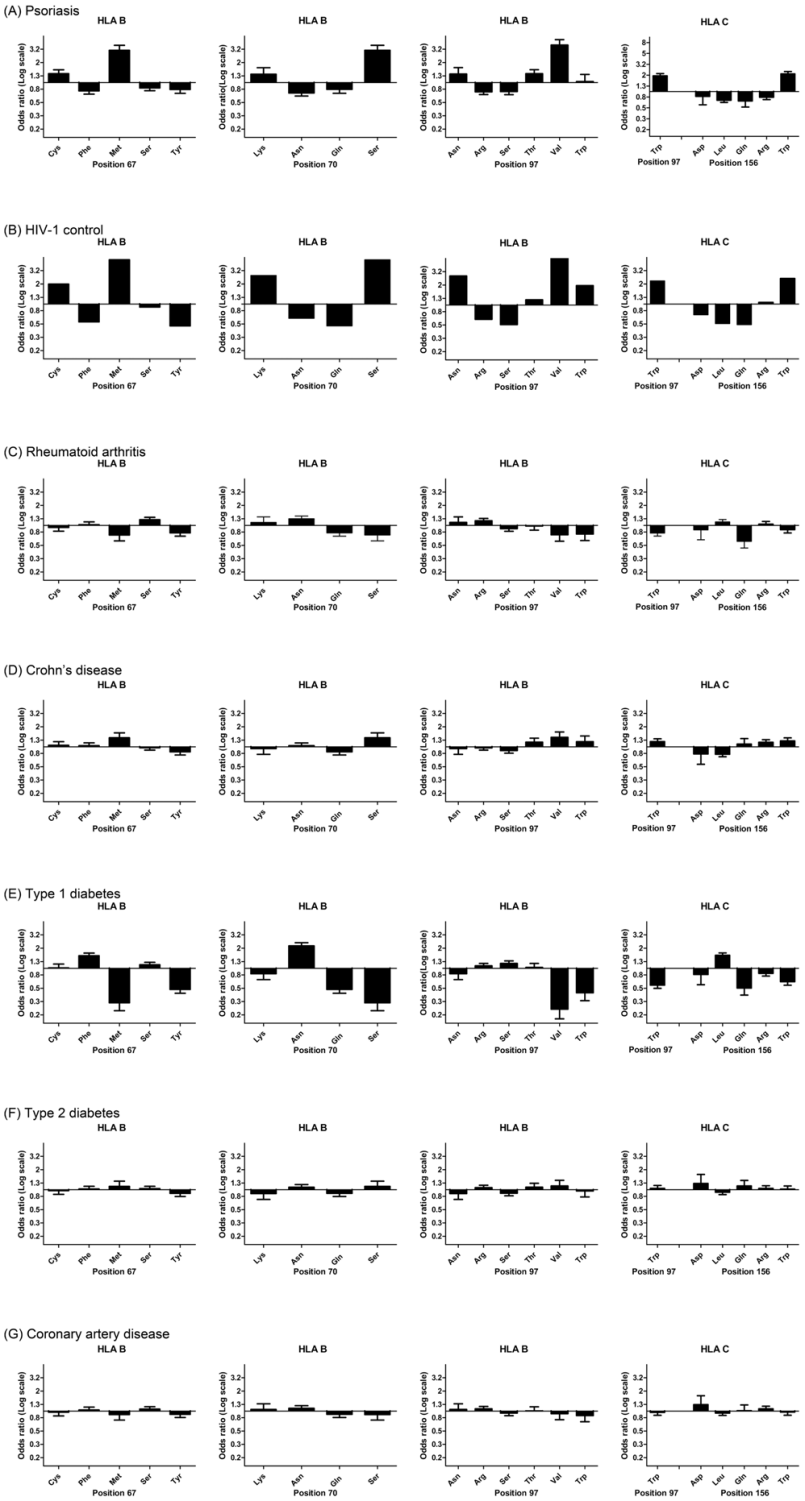
Top 5 HLA amino acid positions associated with psoriasis and comparison with HIV-1 control and other autoimmune or inflammatory diseases. (A) Specific amino acid residues at positions 67, 70, and 97 within HLA-B and positions 97 and 156 within HLA-C are strongly associated with psoriasis susceptibility or protection, where the strength and direction of association are reflected by the odds ratio at each residue. All 5 positions occur in the peptide-binding groove of HLA-B or HLA-C. (B) Comparison of odds ratios to HIV-1 control, in which HLA-B positions 67, 70, and 97 are the top 3 three reported positions [15]. (C–G) Comparison of odds ratios to five other autoimmune or inflammatory diseases from the Wellcome Trust Case-Control Consortium. None of these demonstrate the same degree of similarity as between psoriasis and HIV-1 control. To demonstrate that this similarity cannot be entirely explained by the association of psoriasis with B*57:01 and C*06:02, we conditioned each of these residues on both B*57:01 and C*06:02 and found that Cys^67^, Ser^67^, Lys^70^, Asn^97^, Arg^97^ in HLA-B remained independently associated with psoriasis (all p<5×10^−4^) in the same direction as the association with HIV-1 control. This suggests that the repertoire of peptides bound at HLA-B may be similar between psoriasis patients and HIV-1 controllers.

Specific amino acid positions within the peptide binding groove of HLA class I molecules have been shown to serve as important mediators for the protective and risk effects of individual HLA alleles on HIV-1 control [15]. Namely, amino acid residues at positions 97, 67, and 70 within HLA-B were found to be more highly associated with HIV-1 control than HLA-B*57:01 and each of these amino acid positions was found to serve as a strong predictor of HIV-1 viral load levels in an independent cohort [15]. To determine whether psoriasis is associated with the groups of alleles that are marked by specific amino acids within HLA proteins, we used the official protein sequences [31] assigned to each four-digit HLA allele to perform association testing at each amino acid position within *HLA-A, -B, -C, -DQA1, -DQB1*, and *-DRB1*. As before, the association testing was adjusted for gender, ancestry, and cohort. We found that the five most significant amino acid positions for psoriasis occurred at 3 positions within HLA-B (residue 97 [p = 1.58×10^−53^], residue 67 [p = 4.00×10^−45^], and residue 70 [p = 1.35×10^−40^]) and 2 positions with *HLA-C* (residue 156 [p = 3.89×10^−51^] and residue 97 [p = 4.56×10^−45^]) (Table S4). Each of these 5 positions is located within the peptide-binding groove of the HLA molecule and directly contacts the bound peptide [32]. At each of these positions, we investigated whether the direction of the association signal was consistent between psoriasis patients and HIV-1 controllers. We confirmed that for each position examined, the amino acid residues associated with psoriasis susceptibility were associated with HIV-1 virologic control, and the amino acids associated with a protective effect on psoriasis risk were associated with HIV-1 progression (Figure 1). For example, alleles marked by Asn^97^, Thr^97^, and Val^97^ in HLA-B were associated with psoriasis susceptibility and HIV-1 control while those marked by Arg^97^ and Ser^97^ in HLA-B were associated with psoriasis protection and HIV-1 progression.

To rule out the possibility that the observed similarities between psoriasis and HIV-1 control were the result of systematic bias of the imputation process or general amino acid variability at those positions, we examined whether these 5 amino acids positions were associated with other autoimmune or inflammatory diseases. We examined GWAS data from 5 diseases studied by the Wellcome Trust Case Control Consortium [33]—rheumatoid arthritis, Crohn's disease, type 1 diabetes, type 2 diabetes, and coronary artery disease—and used the same imputation process as performed with psoriasis. We found that none of these diseases displayed the degree of similarity between psoriasis and HIV-1 control when considering the direction and magnitude of the association signal at these amino acid positions (Figure 1). Crohn's disease, which shares some pathophysiological features with psoriasis [34] and is also slightly enriched for HLA-C*06:02 (p = 4.2×10^−5^, OR 1.32) and HLA-B*57:01 (p = 3.68×10^−4^, OR 1.40), showed some similarity to psoriasis and HIV-1 control at these positions, but the magnitude of the association was smaller and several important residues such as Asn^70^ and Asn^97^ in HLA-B and Gln^156^ in HLA-C were not concordant. Interestingly, type 1 diabetics showed the opposite effect at many of these residues (i.e. patients with type 1 diabetes lack HIV-1 control alleles and have an excess of HIV-1 progression alleles), which could support the theory that type 1 diabetes is triggered by a viral infection.

Another important amino acid within HLA-B that may be relevant for HIV-1 progression is position 116, which not only interacts with the carboxy-terminal residues of peptides in the F pocket, but also strongly influences the interaction of HLA class I molecules with the peptide-loading complex [35], [36]. Studies of HLA-B*44:05 and HLA-B*44:02, which only differ at position 116, have shown that B*44:05 (containing tyrosine at position 116, “116Y”) utilizes a tapasin-independent pathway that leads to a less optimal peptide repertoire compared to the tapasin-dependent HLA-B*44:02 (116D) [36]. 116Y is strongly associated with lack of HIV-1 control, p = 1.6×10^−10^, OR = 0.57 [15]. Our data show that 116Y is strongly associated with decreased susceptibility to psoriasis, p = 7.96×10^−17^, OR = 0.66 [0.60–0.73] (Table S4). In our dataset, all *HLA-B* alleles containing 116Y had an odds ratio less than 1.0, including B*07:02 (p = 3.4×10^−6^, OR = 0.71), B*08:01 (p = 0.092, OR = 0.88), B*35:02 (p = 0.038, OR = 0.54), B*40:01 (p = 7.24×10^−6^, OR = 0.60), B*40:02 (p = 0.013, OR = 0.53), and B*51:01 (p = 0.084, OR = 0.82). Thus, protection against psoriasis may be associated with presentation of a less-optimized peptide repertoire.

Together, our data indicate that the genetic similarity between psoriasis patients and HIV-1 controllers extends to specific amino acid residues within class I molecules that mediate peptide binding, influence peptide loading, and which mark viral control or progression. We additionally performed stepwise regression to identify amino acid residues that were independently associated with psoriasis and found Trp^156^ and Ala^24^ in HLA-C; Val^97^, Leu^145^, Cys^67^, and Tyr^99^ in HLA-B; and Gly^107^ in HLA-A to be markers independently associated with psoriasis (Table S5). Similar to HIV-1 control [15], we found that HLA-B positions 97 and 67 remained in the model, while position 70 dropped out due to linkage disequilibrium with positions 97 and 67. However, we caution against an interpretation that the amino acids identified here as independent are necessarily the functional ones. Due to the complex LD patterns between the amino acids, the final output of the stepwise regression model is affected by the starting variable, and potentially functionally significant amino acids can be lost because they are tagged by other residues. For example, HLA-B Gly^62^, part of the α1 helix located in the B-pocket of the peptide binding groove, shows strong independent association with HIV-1 control (p = 4.6×10^−27^, OR = 5.03) [15]. Gly^62^ is also strongly associated with susceptibility to psoriasis (p = 2.03×10^−39^, OR = 3.20) but is in high LD with HLA-B Val^97^ and thus drops out of the final psoriasis model.

### The HIV-1 Protective *HLA-C* 3′UTR Deletion Polymorphism rs67384697 Is Enriched in Psoriasis

Expression levels of *HLA-C* are modulated by the G/- polymorphism rs67384697 located within the 3′ UTR of *HLA-C*, where the presence of the deletion inhibits the binding of the microRNA hsa-miR-148 to the 3′UTR and results in higher HLA-C surface expression [37]. In a multivariate model of HIV-1 control, the deletion allele of rs67384697 has a strong effect on viral control independent of the classical alleles HLA-B*57:01 and HLA-B*27:05, although the high LD of rs67384697 with other *HLA-B* and *HLA-C* alleles makes it difficult to determine whether rs67384697 (high HLA-C expression) is directly mediating this effect, or whether the HLA alleles themselves are causal [37]. Nevertheless, rs67384697 has been proposed to be the functional variant that explains the previously identified protective effect of rs9264942 on HIV-1 control, where rs9264942 is located -35 kb upstream of *HLA-C* and is in moderately high LD with rs67384697 (r^2^ = 0.74). We investigated whether rs67384697 was associated with psoriasis by imputing the deletion genotype of all psoriasis cases and controls. This was made possible by the near perfect LD between *HLA-C* four digit classical alleles and presence or absence of the deletion [Supplementary Table 2 in [37]]. To confirm the validity of our imputation, we sequenced the region of the *HLA-C* 3′UTR containing rs67384697 in a subset of our samples (n = 70) and found a concordance rate of 138/140 alleles (98.6%), indicating the imputation was robust. Using logistic regression and adjusting for sex, ancestry, and cohort, we found that deletion allele of rs67384697 was significantly associated with psoriasis (p = 1.02×10^−29^, OR = 1.72) (Table 5), again confirming the similarity between psoriasis patients and HIV-1 controllers. We found that the association of rs67384697 with psoriasis was largely driven by HLA-C*06:02, since conditioning on HLA-C*06:02 resulted in only a marginally significant p-value for rs67384697 (p = 0.044, OR = 1.12). We note, however, that among all HLA-C allotypes, HLA-C*06:02 shows the highest level of cell surface expression, which could explain, to some extent, its strong association with psoriasis.

**Table 5:** Association of rs67384697 with psoriasis and conditional analysis on HLA-C*06:02 and B*57:01.

Allele	Frequency in cases	Frequency in controls	P value	OR	95% CI
rs67384697 del	0.513	0.398	1.02E-29	1.72	1.57-1.89
Condition on C*06:02			0.044	1.12	1.00-1.25
Condition on B*57:01			3.05E-13	1.46	1.32-1.62

rs67384697, a G/del SNP located in the 3′UTR of HLA-C, has been shown to modulate HLA-C expression levels by affecting the binding of microRNA hsa-miR-148 to its target site [37]. rs67384697 is associated with HIV-1 control independent of the classical HLA-alleles HLA-B*5701 and HLA-B*27:05.

### *KIR3DS1* Plus *HLA-B Bw4-80I* Is a Risk Factor for Psoriasis

Natural killer (NK) cells, a major component of the innate immune system, respond in the early stages of viral infection by producing cytokines and killing infected cells. NK-cell responses are regulated in part by activating and inhibitory killer immunologlubulin-like receptors (KIRs) on NK cells which engage HLA class I molecules on target cells. The activating KIR allele KIR3DS1 on chromosome 19, alone or in combination with its putative *HLA-B* ligand Bw4, has been associated with delayed progression to AIDS and improved HIV-1 outcomes [38]–[43]. The *HLA-B* Bw4 epitope can be identified by the presence of isoleucine or threonine at amino acid position 80, whereas the Bw6 epitope contains asparagine at position 80.

Our HLA data revealed that psoriasis is associated with *HLA-B* alleles carrying the Bw4 epitope (p = 1.28×10^−25^, OR = 1.66, Table S4). The association was stronger for *Bw4-80I* [isoleucine] (p = 8.28×10^−22^, OR = 1.80) than for *Bw4-80T* [threonine] (p = 3.41×10^−4^, OR = 1.22), which is interesting because *Bw4-80I* is thought to have a higher binding affinity for its KIR receptor than *Bw4-80T* [44]. We therefore hypothesized that psoriasis susceptibility might be mediated through activation of NK cells through *KIR3DS1* and its putative partner *HLA-B Bw4-80I*. We genotyped *KIR3DS1* in a subset of our psoriasis samples (n = 397) and compared the results to a healthy control cohort with available *KIR3DS1* and HLA genotypes (n = 282). We found that the presence of the compound genotype *KIR3DS1*+*Bw4-80I* was a strong risk factor for psoriasis (frequency 22.7% in cases vs 6.9% in controls, p = 1.54×10^−7^, OR = 3.92, Table 6). Individuals positive for *KIR3DS1* but lacking *Bw4-80I* had no increased risk for psoriasis (p = 0.63, OR = 0.91), and individuals positive for *Bw4-80I* but lacking *KIR3DS1* had only a borderline increased risk of psoriasis (p = 0.058, OR = 1.53). To our knowledge, this is the first report that the compound genotype *KIR3DS1*+*Bw4-80I* is a strong risk factor for psoriasis susceptibility. This finding is again consistent with our observation that there is significant genetic similarity between psoriasis patients and HIV-1 controllers; however, replication of the *KIR3DS1*+*Bw4-80I* association in additional psoriasis cohorts is warranted.

**Table 6:** Association Testing of KIR3DS1 with Psoriasis.

Genetic variable	Frequency^a^ in cases (%)	Frequency^a^ in controls (%)	P-value^b^	OR	95% CI
KIR3DS1 without Bw4-80I	79/331(23.9)	63/245(25.7)	6.26E-01	0.91	0.61-1.35
Bw4-80I without KIR3DS1	76/331(23.0)	40/245(16.3)	5.84E-02	1.53	0.98-2.40
**KIR3DS1+Bw4-80I** c	**75/331(22.7)**	**17/245(6.9)**	**1.54E-07**	**3.92**	**2.21-7.30**
KIR3DS1	185/397(46.6)	92/282(32.6)	2.68E-04	1.80	1.30-2.51
Bw4-80I	154/339(45.4)	58/247(23.4)	3.76E-08	2.71	1.86-3.98

The combination of KIR3DS1 and HLA-B Bw4-80I has a large effect on psoriasis susceptibility, whereas KIR3DS1 without Bw4-80I, and Bw4-80I without KIR3DS1, have little effect. Thus, the compound genotype KIR3DS1+Bw4-80I drives the individual associations of KIR3DS1 and Bw4-80I with psoriasis (bottom two rows).

a Frequencies of individuals positive for each allele.

b p-values were tested by a two-sided Fisher's exact test (dominant model).

c Logistic regression based conditional association testing of “KIR3DS1+Bw4-80I” on HLA-C*06:02 remained significant at p = 9.99×10^−5^, OR = 3.16 [1.77–5.63], whereas conditional testing of “Bw4-80I without KIR3DS1” on HLA-C*06:02 resulted in a non-significant p-value of 0.579, OR = 1.14 [0.72–1.80]. Thus, Bw4-80I does not have an independent effect on psoriasis susceptibility in the absence of KIR3DS1.

## Discussion

In this study, we followed up on the observation that several of the top SNPs from genome-wide association studies of psoriasis were identical to the top SNPs from genome wide association studies of HIV-1 control. Using imputation of HLA alleles, we found that psoriasis patients are enriched for several of the most significant known genetic variants associated with HIV-1 control: HLA-B*57 and HLA-B*27, which are associated with decreased viral load and increased time to AIDS [14], [15], [24]–[27]; specific amino acid residues at *HLA-B* positions 97, 67, and 70 that are strong markers of HIV-1 controller status and viral load [15]; the deletion SNP rs67384697 which is associated with decreased viral load independent of HLA-B*57 and HLA-B*27 [37]; and the activating *KIR3DS1* allele in combination with *HLA-B Bw4-80I* [42]. Psoriasis patients also demonstrate a significant paucity of HLA alleles and variants associated with HIV-1 disease progression [15], [28], including HLA-B*35 (especially B*35-Px), B*07:02, B*40, C*04:01, C*07, and tyrosine 116 in *HLA-B* associated with sub-optimal peptide loading (Table S2, Table S4). These effects were consistent between the 3 psoriasis cohorts examined in this study, demonstrating that the effects observed were real (Table S6).

An important question to address, however, is whether the structural similarity between HLA alleles in psoriasis and HIV-1 control reflects the same underlying causal variants, or merely a coincidental association due to linkage disequilibrium. Our data suggest that some, but not all, of the observed similarity can be attributed to linkage disequilibrium. Our haplotype analysis shows that both psoriasis patients and HIV-1 controllers are enriched for the same extended haplotype, B*57:01–C*06:02–DQA1*02:01–DQB1*03:03–DRB1*07:01. In the HIV-1 controllers, this haplotype is likely primarily driven by selection for B*57:01, since previous studies have shown that the association of C*06:02 with HIV-1 control is dependent on B*57:01 in Europeans [15] and B*5801 in Africans [45], although one indirect benefit of C*06:02 for HIV-1 control is its high LD (D′ = 1) with the rs67384697 deletion polymorphism. In psoriasis, the haplotype association appears to be driven more by C*06:02 than B*57:01, since C*06:02 remains significant after conditioning on B*57:01 (p = 6.86×10^−39^, OR = 3.04) and a number of C*06:02 haplotypes that do not contain B*57:01 still remain associated with psoriasis (Table 3). In addition, the association of the deletion allele of rs67384697 with psoriasis appears to be largely driven by LD with C*06:02. However, it should be noted that variants in high LD with C*0602 may be contributing to the observed association signal for C*0602. Interestingly, one can take the association signal for C*06:02 in psoriasis and perform stepwise conditioning on all coding amino acids within C*06:02 to demonstrate that the coding residues do not account for the entire association signal (Table S7). Therefore, the association of C*06:02 with psoriasis reflects, in part, other variants in high LD with C*06:02.

Despite the effects of linkage disequilibrium, our data suggest that some HIV-1 control variants indeed contribute independently to psoriasis susceptibility. First, both HLA-B*57:01 and HLA-B*27:05 remain associated with psoriasis after conditioning on C*06:02 (B*57:01 p = 1.43×10^−3^, OR = 1.45; B*27:05 p = 4.83×10^−4^, OR = 1.52) and both remain independently associated with psoriasis in our stepwise regression model (Table 2). A previous analysis of the HLA region in psoriasis by Feng et al. [46] also found that B*57:01 was independent of C*06:02 in Caucasians; moreover, in this study B*57:01 was also found to be independent of C*06:02 in a Chinese psoriasis cohort, which is notable because the LD between C*06:02 and B*57:01 is lower in Asians (D′ = 0.41) compared to Europeans (D′ = 0.90) [47]. Prior studies have also shown that B*27 is a strong risk factor for psoriasis in the subset of patients with psoriatic arthritis, especially those with axial disease [48]–[51]. Second, linkage disequilibrium with C*06:02 does not explain the lower frequency of the HIV-1 progression allele B*35 in psoriasis, nor can it account for the concordance of amino acid residues at *HLA-B* positions 67, 70, and 97 whose association with psoriasis was shown to be independent of HLA-B*57:01 and HLA-C*06:02 (Cys^67^, Ser^67^, Lys^70^, Asn^97^, Arg^97^, see Figure 1. legend). Finally, the provisional association of *KIR3DS1+HLA-B Bw4-80I* with psoriasis cannot be due to linkage disequilibrium, because *KIR3DS1* is located on chromosome 19 which segregates independently of chromosome 6.

Although B*57:01, B*27:05, and possibly B*35 may have independent effects in psoriasis, additional studies are needed to clarify the precise mechanism(s) by which these and other psoriasis-associated HLA alleles contribute to psoriasis susceptibility or protection. The observation that psoriasis patients and HIV-1 controllers display concordant amino acids within the peptide binding groove of HLA-B suggests the possibility that an unknown psoriasis antigen shares homology with HIV-1 epitopes. An alternative possibility is that B*57:01, B*27:05, and B*35 do not restrict antigen presentation in psoriasis, but primarily function through their ability or inability to activate NK cells. We have provisionally shown a strong effect of *KIR3DS1+Bw4-80I* on psoriasis susceptibility, and B*57:01 contains the *Bw4-80I* epitope. The second strongest HLA allele in our stepwise regression model, B*38:01, also contains the *Bw4-80I* epitope. B*27:05 contains the *Bw4-80T* epitope, while protective alleles B*35 and B*40 contain the *Bw6* epitope, which do not serve as ligands for KIR. Previous studies have shown that the activating KIR allele *KIR2DS1* also contributes to psoriasis or psoriatic arthritis susceptibility [52]–[55], supporting the notion that NK cells may play a role in the pathogenesis of psoriasis. Finally, we have discussed the potential role of peptide processing on susceptibility to psoriasis, with the presence of tyrosine at HLA-B position 116 associated with protection against psoriasis, where position 116 is located near the C terminus of the bound peptide. A role for peptide processing influencing psoriasis risk has been previously identified for the gene *ERAP1*, an amino peptidase which regulates the quality of peptides bound to MHC class I molecules through trimming the peptide N terminus [10].

The genetic similarity between psoriasis patients and HIV-1 controllers has interesting implications. On a population level, the data would predict that Caucasian individuals with psoriasis are more likely than Caucasian individuals without psoriasis to be HIV-1 controllers, and HIV-1 controllers are more likely than non-controllers to develop psoriasis. This does not imply that every individual with psoriasis will be an HIV-1 controller, since only a fraction of psoriasis patients will harbor, for example, B*57, and even the presence of B*57 does not guarantee HIV-1 control, as this allele is present in some HIV-1 progressors. Nevertheless, one would expect an enrichment of HIV-1 controllers in the psoriasis population relative to a non-psoriatic population.

Our data also suggest a hypothesis that the existence of psoriasis may represent aberrant activation of evolutionarily-derived viral control alleles [56]. Barreiro et al. have shown that several of the top MHC SNPs associated with both psoriasis and HIV-1 control reside on haplotypes which show strong evidence of recent positive selection in the genome, as evidenced by long haplotypes indicative of rapid expansion of an advantageous allele in the population [57]. Psoriasis could subsequently result from the activation of viral control alleles due to the presence of a psoriasis antigen with sequence homology to HIV-1, or due to other environmental triggers. Although this study has focused on HLA and KIR alleles, other non-MHC psoriasis genes are plausibly associated with host response to viral infection. *ERAP1* is involved in class I peptide processing and demonstrates epistasis with C*06:02 [10] and *IFIH1* encodes a cytoplasmic helicase that mediates induction of interferon response to viral RNA [10], [58]. *TNIP1*, *TNFAIP3*, *TRAF3IP2*, *NFKBIA*, and *REL* are associated with the TNF-α pathway and activation of NF-κB; while *IL23R*, *IL12B*, *IL23A*, and *TYK2* are associated with activation of the Th17 pathway. Psoriasis is characterized by the upregulation of the cytokines IFN-α, IFN-γ, TNF-α, IL-17, IL-22, and IL-23 [59], while TNF-α, IFN-γ, and Th17+ T cells have been shown to be important in HIV-1 controllers [60]–[62].

**Figure 2:**
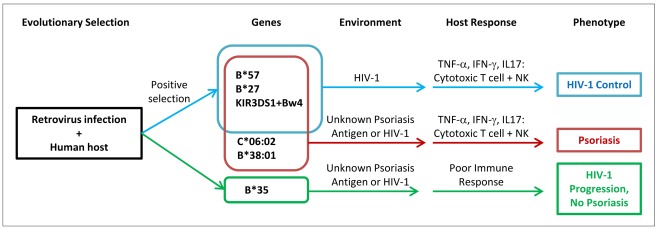
Proposed model of relationship between psoriasis and HIV-1 control. Human ancestors encountered retroviruses similar to HIV-1, leading to positive selection for viral control alleles such as B*57. Individuals who develop psoriasis are enriched for viral control alleles that are aberrantly activated by environmental triggers or unknown skin antigens (possibly sharing homology to HIV-1 epitopes). When individuals with psoriasis become infected with HIV-1, they mount vigorous cytotoxic T cell and natural killer cell immune responses leading to secretion of pro-inflammatory cytokines which worsens the psoriasis. The genetic determinants for psoriasis and HIV-1 control are overlapping, but not identical.

The enrichment of viral control alleles in psoriasis patients may also help explain the psoriasis HIV-1 paradox (Figure 2). Psoriasis patients are more likely to harbor alleles such as HLA-B*57, HLA-B*27, HLA-C*06 (in high LD with the *HLA-C* 3′UTR deletion polymorphism), and *KIR3DS1+Bw4-80I*, theoretically resulting in vigorous cytotoxic T cell and NK cell responses upon infection with HIV-1 virus. The pro-inflammatory environment created by these anti-viral responses, resulting in the production of cytokines such as TNF-α and IFN-γ, would worsen the psoriasis. In addition, if the psoriasis antigen had sequence homology to HIV-1, then antigen specific immune responses directed against HIV-1 might cross-react with the psoriasis antigen and also flare the psoriasis. In either case, reduction of viral load and removal of the antigenic trigger through treatment with anti-retroviral therapies would improve the psoriasis, which is indeed seen clinically [17]. This explanatory model is consistent with several observational studies that patients with severe psoriasis and HIV-1 infection tend to carry the HLA-C*06 and HLA-B*27 alleles [63], [64], because such alleles would trigger the vigorous immune response associated with exacerbation of psoriasis.

The data presented here with psoriasis and HIV-1 control illustrate the delicate balance of the human immune response, in which processes that safeguard the body against pathogens may also engage deleterious inflammatory responses. A similar example occurs with a genetic variant in the *SH2B3* gene which may be protective against bacterial infection but which increases susceptibility to celiac disease, an autoimmune disease of the gut resulting from gluten intolerance [65]. Another example can be seen with the identification of genetic variants in immune function genes that increase the risk of sepsis, a systemic inflammatory response to infection which can lead to death [66], [67].

In summary, using a large dataset of psoriasis cases and controls, we have shown that psoriasis patients and HIV-1 controllers share a high degree of similarity at their HLA loci. While some of this similarity is attributable to linkage disequilibrium, we present evidence that much of the similarity may be attributable to shared biological mechanisms including activation of natural killer cells, specificity of antigen presentation, and use of optimal MHC class I peptide processing. The genetic similarity between psoriasis and HIV-1 control suggests the possibility that psoriasis represents aberrant activation of pathways associated with anti-viral immunity. If this hypothesis is true, then the study of the biological pathways active in psoriasis may provide new therapeutic insights for the treatment of HIV-1.

## Materials and Methods

### Study Subjects

The study population and source are shown in Table S1. Two independent genome-wide association scan datasets were used as cohort 1 and cohort 2 in the present study. All cases and controls were of European descent. More details on subject characteristics and recruitment can be found in Liu et al. [8] and Nair et al. [9]. Only the subjects whose HLA alleles were successfully imputed (see below) were included in our analysis. In cohort 3, 169 psoriasis cases recruited from Washington University, St. Louis were directly typed for the class I HLA alleles by combining locus-specific amplification with hybridization of sequence-specific oligonucleotide probes as described in [68]; 1,711 control samples of European ancestry were obtained from studies 66 and 67 of illumina iControlDB. There was no overlap between the subjects among the three cohorts. Informed consent was obtained from each participant.

### HIV Cohorts

Data generated by this study were primarily compared against a published genome-wide association study of HIV-1 control involving 516 HIV controllers of European ancestry (viral load <2,000 RNA copies/ml by three measurements over at least 12 months without antiviral therapy) and 1,196 controls (treatment-naïve chronically infected individuals with advanced disease, median viral load 61,698 copies/ml) [15]. HLA haplotype analysis was performed on 214 Caucasian HIV-1 infected individuals (52 HIV-1 controllers, 162 non-controllers) from the SCOPE cohort (Study of the Consequences of the Protease Inhibitor Era), whose HLA class I and II alleles had been previously directly genotyped. SCOPE HIV-1 controllers were antiretroviral therapy-naïve subjects who had at least one year duration of documented plasma HIV RNA below 2,000 copies/ml, while SCOPE non-controllers were subjects who had at least one documented viral load above 10,000 copies/ml.

### WTCCC Data

The Wellcome Trust Case-Control Consortium data were obtained from the WTCCC official website (http://www.wtccc.org.uk/). In this study, we used Affymetrix 500 K genotyping data from approximately 2,000 samples from each of five diseases (rheumatoid arthritis, Crohn's disease, type 1 diabetes, type 2 diabetes, and coronary artery disease) and 3,000 shared control samples from the 1958 birth cohort (58C) and the National Blood Service (NBS). More details about these samples are described elsewhere [33].

### KIR Analysis Cohorts

KIR3DS1 typing was performed on 397 psoriasis subjects from cohorts 2 and 3 described above. Control HLA and KIR3DS1 data were obtained from 282 healthy Caucasian blood donors from the Carrington laboratory.

### Imputation and Validation of Classical HLA Alleles

The program HLA*IMP [23] was used to impute HLA loci -A, -B, -C, -DQA1, -DQB1 and -DRB1 to 4-digit resolution in our genome-wide SNP datasets. Individuals or SNPs with a missing data frequency above 0.20 were excluded as recommended in the software manual. A call threshold of 0.7 on the modes of the posterior HLA type distributions was employed, which represents a good compromise between accuracy and call rate as suggested by the author. Imputation accuracy was assessed by comparing the imputed HLA alleles to directly typed HLA class I alleles in a subset of our samples (n = 98), comprising 42 samples imputed from Illumina 300 K SNP data and 56 samples imputed from Perlegen SNP data. We found that HLA*IMP produces highly accurate HLA type imputations at HLA class I loci at the 4-digit level. The concordance for the Illumina 300 K platform was 244/252 alleles (96.8%) and the concordance for the Perlegen platform was 322/329 alleles (97.9%), for an overall concordance rate of 566/581 alleles (97.4%). To examine the imputation accuracy of infrequent/rare HLA alleles, we identified all HLA class I alleles with a population frequency of less than 5% in individuals of European descent, according to the online database: http://www.allelefrequencies.net. We then examined the accuracy of imputation at the 4 digit level for these infrequent/rare HLA alleles in our subjects for whom we had both directly genotyped HLA alleles and imputed HLA alleles. The concordance of HLA alleles with frequency <5% was 95/102 alleles (93.1%) for the Illumina 300 K platform and 116/122 alleles (95.1%) for the Perlegen platform, for an overall concordance rate of 211/224 alleles (94.2%). However, our manuscript excludes HLA alleles with frequency less than 1%. For HLA alleles with frequency greater than 1% but less than 5%, the concordance was 78/79 alleles (98.7%) for Illumina 300 K and 99/100 alleles (99.0%) for Perlegen, for an overall concordance rate of 177/179 alleles (98.9%).

### Association Testing and Adjustment for Covariates

Additive logistic regression models in PLINK [69] were used for most of the association tests, except for the HLA haplotype association tests. To account for potential population stratification or admixture in these samples, principal component analyses (PCA) was performed using the EIGENSTRAT [70]. Seven PCs in cohort 1 and ten PCs in cohort 2 were used for ancestry adjustment, based on leveling off of the PCA scree plot. The principal component score for each individual was included as a covariate in all models along with cohort and gender in logistic regression models. Multivariate logistic regression was performed in R software package (http://www.r-project.org/). To examine the consistency of association signals seen in the 3 cohorts used, a heterogeneity index was calculated using the meta-analysis module in PLINK.

### Stepwise Regression Modeling of Independent HLA Alleles

Conditional and stepwise logistic regression was performed using the ‘condition’ function in PLINK to determine whether independent effects existed. The method begins with an empty model to which variables are added in an iterative process as described by Barcellos et al [71]. Briefly, starting with HLA-C*06:02 which exhibits the strongest association with psoriasis, we conditioned candidate HLA alleles on C*06:02 to determine the next most significant independent effect. For the model including both class I and class II alleles, the iterative process completes when no candidate allele demonstrates p<0.0006, which corresponds to the Bonferroni correction for the 88 HLA candidate alleles with MAF>1% tested. For the model including only class I alleles, the iterative process completes when no candidate allele demonstrates p<0.00096, which corresponds to the Bonferroni correction for the 52 class I candidate alleles with MAF>1% tested.

### Association Testing of Amino Acid Positions (Including WTCCC Diseases)

The amino acid sequence of all HLA alleles is completely determined by the HLA type at four-digit resolution. We used the official amino acids sequences defined for known HLA alleles [31] and our imputed HLA allele data to determine the frequency of amino acid residues in cases and controls. HLA amino acids residues were tested for association using a logistic regression model that corrects for population substructure, gender and cohort using PLINK. For amino acid positions with >2 alleles, the omnibus test in the conditional haplotype analysis module in PLINK was used to determine a single p-value for all alleles at that position.

### Stepwise Regression Modeling of Amino Acid Residues

We performed stepwise logistic regression in PLINK to determine the amino acid residues that were independently associated with psoriasis, using the same approach as done with the HLA alleles. For the amino acid analysis, the algorithm completes when no remaining candidate residue has p<0.0001, which corresponds to the Bonferroni correction for the 480 HLA amino acid residues with MAF>1% tested. For the stepwise regression modeling of HLA-C 06:02 association signal (Table S7), the algorithm completes when no candidate residue has p<0.0006(0.05/87).

### Imputation and Validation of rs67384697 Deletion Polymorphism

Since there is strong linkage disequilibrium between specific HLA-C alleles and rs67384697 [Supplementary Table 2 in [37]], we were able to determine the rs67384697 genotype of all subjects using their HLA-C four digit classical alleles. To ensure the accuracy of our imputation, we directly sequenced (ABI 3730 DNA analyzer, Quintara Biosciences, Berkeley, CA) the region of the HLA-C 3′UTR containing rs67384697 in a subset of our samples (n = 70) and determined a high concordance rate of 138/140 alleles (98.6%). The following primers were used for sequencing of genomic DNA samples: forward 5′-gtgagattctggggagctga and reverse 5′-gaacagcaactaggcacagg as specified in [37].

### Haplotype Analysis

Arlequin V3.5, based on the EM algorithm, was used to estimate the frequency of HLA allele haplotypes in our psoriasis cohorts and the SCOPE HIV cohort. To ensure the accuracy of haplotype construction, we compared the haplotype frequencies generated by Arlequin to the frequencies obtained by direct counting of the phased HLA alleles output from HLA*IMP, and found the two methods to yield nearly identical results. Haplotype frequencies were tested for statistically significant differences between case and control groups using the Chi Square test or Fisher's exact test in the R software package.

### KIR Genotyping

*KIR3DS1* genotyping was performed by using multiplex PCR-SSP (sequence-specific priming) according to Kulkarni et al with other minor modifications [72]. Briefly, each reaction contained 15 ng of DNA, 200 µM dNTP, 1.5 mM MgCl2, 0.5 µl 10× PCR buffer, 1 µM of each primer for *KIR3DS1* and *KIR3DL1* and 0.8 µM of each primer for *HLA-DRB1*, and 0.025 µl of Platinum Taq polymerase (Invitrogen, Carlsbad, CA) in a 5 µL final volume. The polymerase chain reaction (PCR) conditions were: 3 min at 94°C; 5 cycles of 15 s at 94°C, 15 s at 65°C, 30 s at 72°C; 25 cycles of 15 s at 94°C, 15 s at 60°C, 30 s at 72°C; 4 cycles of 15 s at 94°C, 1 min at 55°C, 2 min at 72°C followed by a final 7 min extension step at 72°C. To confirm the accuracy of the results, samples were replicated using a second set of *KIR3DS1* and *KIR3DL1* primers from [73]. Phenotype frequencies for the presence of each gene were estimated by direct counting. Frequency differences between psoriasis and control groups were tested for significance by two-sided Fisher's exact test.

## Supporting Information

Table S1 Study population and source. GAIN: The Genetic Association Information Network. WashU: Washington University in St. Louis. UCSF: University of California San Francisco. Illumina iControlDB: an online database of genotype and phenotype data from individuals that can be used as controls in association studies.
(DOC)

Table S2 Association results for the imputed classical HLA alleles at 4-digit resolution. P values and ORs were adjusted for ancestry, gender, and cohort. The last two columns show the p values and ORs after conditioning on HLA-C*06:02. Only alleles with frequency greater than 1% in the control group were analyzed.
(DOC)

Table S3 Association results for the imputed classical HLA alleles at 2-digit resolution. P values and ORs were adjusted for ancestry, gender, and cohort. The last two columns show the p values and ORs after conditioning on HLA-C*06. Only alleles with frequency greater than 1% in the control group were analyzed.
(DOC)

Table S4 Association results for the imputed amino acid residues in each of the classical HLA loci. P values and ORs were adjusted for ancestry, gender, and cohort. Only residues with frequency greater than 1% in the control group were analyzed.
(DOC)

Table S5 Amino acid residues identified by stepwise logistic regression as independently associated with psoriasis.
(DOC)

Table S6 Comparison of p-values and odds ratios (ORs) of the top HLA alleles, amino acid residues, and HLA-C 3′ UTR deletion SNP in the 3 psoriasis cohorts used for this study. I^2^ represents the proportion of variation that is due to heterogeneity. Roughly, I^2^ values of 25, 50, and 75 indicate low, moderate, and high heterogeneity. Most variants demonstrate low heterogeneity with a consistent direction of association across the 3 cohorts.
(DOC)

Table S7 Stepwise conditioning of HLA-C*06:02 association signal on the coding amino acids of HLA-C*06:02. The significant residual association indicates that the association of HLA-C*06:02 with psoriasis cannot be entirely explained by coding differences between HLA-C*06:02 and other HLA-C alleles. Therefore, the association of HLA-C*06:02 with psoriasis likely reflects the contribution of other variants in LD with HLA-C*06:02 such as HLA-B*57:01.
(DOC)
