# Supplementary material for: Psoriasis Patients Are Enriched for Genetic Variants That Protect against HIV-1 Disease
Source: PLoS Genet. 2012 Feb 16;8(2):e1002514. doi: 10.1371/journal.pgen.1002514 (PMC3343879; doi:10.1371/journal.pgen.1002514)
Supplement: Table S1 — Study population and source. GAIN: The Genetic Association Information Network. WashU: Washington University in St. Louis. UCSF: University of California San Francisco. Illumina iControlDB: an online database of genotype and phenotype data from individuals that can be used as controls in association studies. (DOC) [file pgen.1002514.s001.doc]

**Table S1. Study population and source.** GAIN: The Genetic Association Information Network. WashU: Washington University in St. Louis. UCSF: University of California San Francisco. Illumina iControlDB: an online database of genotype and phenotype data from individuals that can be used as controls in association studies.

| Cohort | Case-control samples source | N (Psoriasis Case) | N (Control) | Genotyping platform |
| --- | --- | --- | --- | --- |
| 1 | GAIN GWAS [9] | 1348 | 1368 | Perlegen Sciences |
| 2 | WashU/UCSF GWAS [8] | 210 | 502 | Hapmap 300k BeadChip |
| 3 | WashU | 169 | NA | Direct HLA typing |
| 3 | Illumina iControlDB | NA | 1711 | Hapmap 550k BeadChip |
|  | **Total** | **1727** | **3581** | _ |
