# Supplementary material for: Psoriasis Patients Are Enriched for Genetic Variants That Protect against HIV-1 Disease
Source: PLoS Genet. 2012 Feb 16;8(2):e1002514. doi: 10.1371/journal.pgen.1002514 (PMC3343879; doi:10.1371/journal.pgen.1002514)
Supplement: Table S2 — Association results for the imputed classical HLA alleles at 4-digit resolution. P values and ORs were adjusted for ancestry, gender, and cohort. The last two columns show the p values and ORs after conditioning on HLA-C*06:02. Only alleles with frequency greater than 1% in the control group were analyzed. (DOC) [file pgen.1002514.s002.doc]

**Table S2. Association results for the imputed classical HLA alleles at 4-digit resolution.** P values and ORs were adjusted for ancestry, gender, and cohort. The last two columns show the p values and ORs after conditioning on HLA-C*06:02. Only alleles with frequency greater than 1% in the control group were analyzed.

| Allele | Frequency in cases | Frequency in controls | P-value | OR | 95% CI | Condition on C*06:02 | |
| --- | --- | --- | --- | --- | --- | --- | --- |
|  |  |  |  |  |  | P-value | OR |
| A*01:01 | 0.215 | 0.165 | 1.98E-08 | 1.40 | 1.25-1.58 | 8.70E-02 | 1.12 |
| A*02:01 | 0.298 | 0.254 | 5.19E-04 | 1.20 | 1.08-1.33 | 2.00E-04 | 1.23 |
| A*02:05 | 0.011 | 0.012 | 4.85E-01 | 1.17 | 0.75-1.84 | 5.42E-01 | 0.86 |
| A*03:01 | 0.115 | 0.136 | 7.01E-04 | 0.78 | 0.68-0.90 | 2.39E-02 | 0.84 |
| A*11:01 | 0.054 | 0.065 | 1.18E-01 | 0.85 | 0.70-1.04 | 8.98E-01 | 0.99 |
| A*23:01 | 0.015 | 0.024 | 3.16E-02 | 0.67 | 0.47-0.97 | 5.25E-02 | 0.68 |
| A*24:02 | 0.073 | 0.092 | 6.93E-02 | 0.85 | 0.72-1.01 | 1.45E-01 | 0.87 |
| A*25:01 | 0.015 | 0.019 | 1.60E-01 | 0.77 | 0.54-1.11 | 9.54E-01 | 0.99 |
| A*26:01 | 0.037 | 0.033 | 7.69E-01 | 0.96 | 0.74-1.25 | 7.62E-01 | 1.04 |
| A*29:02 | 0.032 | 0.037 | 1.84E-01 | 0.84 | 0.65-1.09 | 3.27E-01 | 0.87 |
| A*30:01 | 0.026 | 0.016 | 2.54E-05 | 2.04 | 1.46-2.84 | 5.19E-01 | 0.89 |
| A*31:01 | 0.03 | 0.032 | 3.02E-02 | 0.75 | 0.58-0.97 | 5.79E-01 | 0.92 |
| A*32:01 | 0.026 | 0.04 | 6.30E-04 | 0.63 | 0.49-0.82 | 1.28E-02 | 0.71 |
| A*68:01 | 0.028 | 0.034 | 7.89E-03 | 0.70 | 0.54-0.91 | 3.44E-01 | 0.88 |
| B*07:02 | 0.098 | 0.122 | 3.40E-06 | 0.71 | 0.61-0.82 | 1.24E-01 | 0.89 |
| B*08:01 | 0.100 | 0.109 | 9.29E-02 | 0.88 | 0.76-1.02 | 7.23E-02 | 1.16 |
| B*13:02 | 0.063 | 0.025 | 6.20E-17 | 2.77 | 2.18-3.51 | 8.00E-01 | 0.97 |
| B*14:02 | 0.031 | 0.03 | 1.89E-01 | 1.20 | 0.91-1.59 | 1.02E-02 | 1.46 |
| B*15:01 | 0.053 | 0.056 | 3.00E-02 | 0.80 | 0.65-0.98 | 5.14E-01 | 0.93 |
| B*18:01 | 0.036 | 0.056 | 1.77E-04 | 0.64 | 0.51-0.81 | 3.22E-02 | 0.77 |
| B*27:05 | 0.049 | 0.035 | 1.59E-02 | 1.32 | 1.05-1.66 | 4.83E-04 | 1.52 |
| B*35:01 | 0.043 | 0.052 | 5.86E-03 | 0.74 | 0.60-0.92 | 3.68E-01 | 0.90 |
| B*35:02 | 0.005 | 0.014 | 3.80E-02 | 0.54 | 0.30-0.97 | 1.07E-01 | 0.61 |
| B*35:03 | 0.005 | 0.016 | 3.17E-03 | 0.42 | 0.24-0.75 | 1.92E-02 | 0.49 |
| B*37:01 | 0.026 | 0.014 | 6.89E-05 | 1.94 | 1.40-2.69 | 7.01E-02 | 0.72 |
| B*38:01 | 0.035 | 0.023 | 1.12E-04 | 1.78 | 1.33-2.38 | 2.69E-07 | 2.21 |
| B*39:01 | 0.022 | 0.012 | 3.64E-03 | 1.70 | 1.19-2.44 | 1.43E-04 | 2.07 |
| B*40:01 | 0.038 | 0.052 | 7.24E-06 | 0.60 | 0.48-0.75 | 4.94E-03 | 0.72 |
| B*40:02 | 0.007 | 0.013 | 1.27E-02 | 0.53 | 0.32-0.87 | 8.49E-02 | 0.63 |
| B*44:02 | 0.072 | 0.088 | 2.91E-03 | 0.77 | 0.65-0.92 | 5.94E-01 | 0.95 |
| B*44:03 | 0.032 | 0.049 | 8.27E-04 | 0.66 | 0.52-0.84 | 4.11E-02 | 0.77 |
| B*49:01 | 0.011 | 0.018 | 7.51E-02 | 0.69 | 0.45-1.04 | 4.52E-01 | 0.85 |
| B*50:01 | 0.015 | 0.011 | 2.15E-01 | 1.30 | 0.86-1.96 | 4.93E-04 | 0.47 |
| B*51:01 | 0.04 | 0.053 | 8.39E-02 | 0.82 | 0.66-1.03 | 9.28E-01 | 1.01 |
| B*55:01 | 0.023 | 0.016 | 1.07E-01 | 1.3 | 0.94-1.80 | 6.81E-03 | 1.58 |
| B*57:01 | 0.125 | 0.039 | 5.50E-42 | 3.61 | 3.00-4.35 | 1.43E-03 | 1.45 |
| C*01:02 | 0.035 | 0.034 | 8.62E-01 | 1.02 | 0.80-1.31 | 2.63E-01 | 1.16 |
| C*02:02 | 0.039 | 0.042 | 7.74E-01 | 0.97 | 0.76-1.22 | 1.67E-01 | 1.19 |
| C*03:03 | 0.054 | 0.05 | 6.65E-01 | 0.96 | 0.78-1.17 | 2.30E-01 | 1.14 |
| C*03:04 | 0.056 | 0.068 | 7.36E-06 | 0.65 | 0.54-0.79 | 1.48E-02 | 0.79 |
| C*04:01 | 0.074 | 0.12 | 2.21E-08 | 0.63 | 0.54-0.74 | 2.10E-03 | 0.77 |
| C*05:01 | 0.067 | 0.081 | 6.72E-03 | 0.78 | 0.66-0.94 | 4.45E-01 | 0.93 |
| C*06:02 | 0.253 | 0.098 | 2.91E-77 | 3.57 | 3.12-4.08 | NA | NA |
| C*07:01 | 0.124 | 0.159 | 4.29E-04 | 0.79 | 0.69-0.90 | 6.18E-01 | 1.04 |
| C*07:02 | 0.11 | 0.128 | 9.03E-05 | 0.76 | 0.66-0.87 | 5.96E-01 | 0.96 |
| C*07:04 | 0.013 | 0.016 | 2.24E-01 | 0.79 | 0.54-1.15 | 5.31E-01 | 1.13 |
| C*08:02 | 0.036 | 0.039 | 8.22E-01 | 1.03 | 0.81-1.31 | 1.00E-01 | 1.24 |
| C*12:03 | 0.069 | 0.06 | 2.16E-02 | 1.25 | 1.03-1.51 | 1.32E-05 | 1.55 |
| C*14:02 | 0.013 | 0.011 | 2.44E-01 | 1.29 | 0.84-1.97 | 4.13E-02 | 1.58 |
| C*15:02 | 0.015 | 0.024 | 1.31E-02 | 0.65 | 0.46-0.91 | 2.22E-01 | 0.8 |
| C*16:01 | 0.023 | 0.036 | 1.65E-03 | 0.64 | 0.48-0.84 | 7.32E-02 | 0.77 |
| C*17:01 | 0.006 | 0.014 | 8.47E-03 | 0.49 | 0.28-0.83 | 8.42E-02 | 0.61 |
| DQA1*01:01 | 0.115 | 0.139 | 7.51E-03 | 0.81 | 0.70-0.95 | 6.62E-02 | 0.86 |
| DQA1*01:02 | 0.165 | 0.197 | 2.53E-03 | 0.82 | 0.72-0.93 | 5.73E-01 | 0.96 |
| DQA1*01:03 | 0.06 | 0.062 | 3.31E-01 | 0.9 | 0.72-1.12 | 9.52E-01 | 1.01 |
| DQA1*02:01 | 0.256 | 0.152 | 3.40E-24 | 1.99 | 1.74-2.27 | 1.73E-02 | 1.21 |
| DQA1*03:01 | 0.166 | 0.166 | 4.01E-01 | 0.94 | 0.82-1.08 | 3.82E-01 | 1.07 |
| DQA1*04:01 | 0.02 | 0.028 | 9.48E-02 | 0.74 | 0.53-1.05 | 1.00E-01 | 0.73 |
| DQA1*05:01 | 0.219 | 0.257 | 2.45E-03 | 0.83 | 0.73-0.94 | 8.61E-01 | 0.99 |
| DQB1*02:01 | 0.096 | 0.104 | 3.37E-01 | 0.92 | 0.78-1.09 | 8.71E-02 | 1.17 |
| DQB1*02:02 | 0.101 | 0.084 | 2.11E-02 | 1.23 | 1.03-1.46 | 1.02E-01 | 0.85 |
| DQB1*03:01 | 0.188 | 0.208 | 1.12E-01 | 0.9 | 0.79-1.03 | 9.05E-01 | 0.99 |
| DQB1*03:02 | 0.107 | 0.099 | 9.62E-01 | 1 | 0.85-1.19 | 1.64E-01 | 1.13 |
| DQB1*03:03 | 0.118 | 0.05 | 1.18E-22 | 2.69 | 2.21-3.29 | 2.47E-03 | 1.41 |
| DQB1*04:02 | 0.027 | 0.034 | 1.09E-01 | 0.78 | 0.58-1.06 | 1.11E-01 | 0.77 |
| DQB1*05:01 | 0.103 | 0.119 | 3.46E-02 | 0.84 | 0.72-0.99 | 1.15E-01 | 0.87 |
| DQB1*05:02 | 0.023 | 0.015 | 1.90E-03 | 1.87 | 1.26-2.77 | 1.93E-04 | 2.23 |
| DQB1*05:03 | 0.034 | 0.04 | 7.99E-02 | 0.79 | 0.60-1.03 | 2.33E-01 | 0.84 |
| DQB1*06:02 | 0.116 | 0.132 | 6.01E-02 | 0.87 | 0.74-1.01 | 6.99E-01 | 1.03 |
| DQB1*06:03 | 0.056 | 0.062 | 2.33E-01 | 0.88 | 0.70-1.09 | 6.85E-01 | 0.95 |
| DQB1*06:04 | 0.019 | 0.037 | 1.46E-05 | 0.49 | 0.35-0.68 | 1.02E-04 | 0.51 |
| DRB1*01:01 | 0.068 | 0.087 | 1.07E-02 | 0.78 | 0.64-0.94 | 9.32E-02 | 0.84 |
| DRB1*01:02 | 0.019 | 0.012 | 1.34E-01 | 1.39 | 0.90-2.13 | 1.02E-01 | 1.46 |
| DRB1*01:03 | 0.017 | 0.015 | 5.39E-01 | 1.14 | 0.75-1.72 | 2.15E-01 | 1.32 |
| DRB1*03:01 | 0.107 | 0.123 | 4.97E-02 | 0.85 | 0.72-1.00 | 5.26E-01 | 1.06 |
| DRB1*04:01 | 0.09 | 0.087 | 7.54E-01 | 1.03 | 0.86-1.23 | 4.96E-01 | 1.07 |
| DRB1*04:04 | 0.036 | 0.045 | 6.41E-02 | 0.78 | 0.60-1.02 | 9.83E-01 | 1 |
| DRB1*07:01 | 0.246 | 0.149 | 1.98E-20 | 1.9 | 1.66-2.18 | 1.99E-01 | 1.11 |
| DRB1*08:01 | 0.024 | 0.035 | 2.82E-02 | 0.7 | 0.51-0.96 | 8.35E-02 | 0.74 |
| DRB1*11:01 | 0.048 | 0.054 | 6.00E-01 | 0.94 | 0.73-1.20 | 9.81E-01 | 1 |
| DRB1*11:04 | 0.014 | 0.022 | 1.44E-01 | 0.73 | 0.48-1.11 | 1.16E-01 | 0.69 |
| DRB1*12:01 | 0.012 | 0.021 | 2.34E-03 | 0.51 | 0.33-0.79 | 5.22E-02 | 0.64 |
| DRB1*13:01 | 0.057 | 0.063 | 1.63E-01 | 0.85 | 0.68-1.07 | 5.82E-01 | 0.94 |
| DRB1*13:02 | 0.028 | 0.049 | 3.93E-05 | 0.55 | 0.42-0.73 | 2.86E-04 | 0.57 |
| DRB1*13:03 | 0.013 | 0.013 | 9.39E-01 | 0.98 | 0.62-1.56 | 2.32E-01 | 1.35 |
| DRB1*14:01 | 0.03 | 0.03 | 9.40E-01 | 0.99 | 0.72-1.35 | 5.63E-01 | 1.1 |
| DRB1*15:01 | 0.117 | 0.136 | 3.45E-02 | 0.85 | 0.72-0.99 | 8.18E-01 | 1.02 |
| DRB1*16:01 | 0.019 | 0.011 | 8.48E-04 | 2.2 | 1.39-3.51 | 1.51E-04 | 2.57 |
