# Supplementary material for: Psoriasis Patients Are Enriched for Genetic Variants That Protect against HIV-1 Disease
Source: PLoS Genet. 2012 Feb 16;8(2):e1002514. doi: 10.1371/journal.pgen.1002514 (PMC3343879; doi:10.1371/journal.pgen.1002514)
Supplement: Table S3 — Association results for the imputed classical HLA alleles at 2-digit resolution. P values and ORs were adjusted for ancestry, gender, and cohort. The last two columns show the p values and ORs after conditioning on HLA-C*06. Only alleles with frequency greater than 1% in the control group were analyzed. (DOC) [file pgen.1002514.s003.doc]

**Table S3. Association results for the imputed classical HLA alleles at 2-digit resolution.** P values and ORs were adjusted for ancestry, gender, and cohort. The last two columns show the p values and ORs after conditioning on HLA-C*06. Only alleles with frequency greater than 1% in the control group were analyzed.

| HLA Allele | Frequency in cases | Frequency in controls | P-value | OR | 95% CI | Condition on C*06 | |
| --- | --- | --- | --- | --- | --- | --- | --- |
|  |  |  |  |  |  | P-value | OR |
| A*01 | 0.216 | 0.165 | 8.81E-09 | 1.42 | 1.26-1.60 | 5.77E-02 | 1.13 |
| A*02 | 0.312 | 0.271 | 2.40E-04 | 1.21 | 1.09-1.34 | 2.23E-04 | 1.23 |
| A*03 | 0.116 | 0.136 | 8.40E-04 | 0.79 | 0.68-0.91 | 2.21E-02 | 0.84 |
| A*11 | 0.054 | 0.065 | 1.29E-01 | 0.86 | 0.70-1.05 | 9.05E-01 | 0.99 |
| A*23 | 0.015 | 0.024 | 3.37E-02 | 0.68 | 0.47-0.97 | 5.53E-02 | 0.69 |
| A*24 | 0.073 | 0.092 | 7.23E-02 | 0.85 | 0.72-1.01 | 1.64E-01 | 0.88 |
| A*25 | 0.015 | 0.019 | 1.66E-01 | 0.78 | 0.54-1.11 | 9.66E-01 | 0.99 |
| A*26 | 0.037 | 0.033 | 7.84E-01 | 0.96 | 0.74-1.25 | 7.19E-01 | 1.05 |
| A*29 | 0.032 | 0.037 | 2.37E-01 | 0.86 | 0.66-1.11 | 3.89E-01 | 0.89 |
| A*30 | 0.033 | 0.029 | 8.89E-03 | 1.44 | 1.10-1.90 | 3.97E-01 | 0.88 |
| A*31 | 0.030 | 0.032 | 3.19E-02 | 0.75 | 0.58-0.98 | 5.89E-01 | 0.93 |
| A*32 | 0.026 | 0.040 | 6.96E-04 | 0.63 | 0.49-0.82 | 1.48E-02 | 0.71 |
| A*68 | 0.037 | 0.043 | 3.97E-02 | 0.78 | 0.62-0.99 | 7.90E-01 | 0.97 |
| B*07 | 0.098 | 0.122 | 3.97E-06 | 0.71 | 0.61-0.82 | 1.29E-01 | 0.89 |
| B*08 | 0.100 | 0.109 | 1.01E-01 | 0.88 | 0.76-1.03 | 6.89E-02 | 1.16 |
| B*13 | 0.063 | 0.025 | 5.12E-17 | 2.78 | 2.19-3.53 | 8.44E-01 | 0.97 |
| B*14 | 0.037 | 0.041 | 8.63E-01 | 1.02 | 0.80-1.31 | 1.00E-01 | 1.24 |
| B*15 | 0.060 | 0.066 | 3.38E-02 | 0.81 | 0.67-0.98 | 7.24E-01 | 0.96 |
| B*18 | 0.036 | 0.056 | 1.99E-04 | 0.65 | 0.51-0.81 | 3.40E-02 | 0.77 |
| B*27 | 0.050 | 0.037 | 1.13E-02 | 1.34 | 1.07-1.67 | 2.89E-04 | 1.54 |
| B*35 | 0.056 | 0.093 | 3.20E-06 | 0.65 | 0.54-0.78 | 6.39E-03 | 0.77 |
| B*37 | 0.027 | 0.014 | 6.17E-05 | 1.95 | 1.41-2.71 | 7.76E-02 | 0.73 |
| B*38 | 0.035 | 0.023 | 9.93E-05 | 1.79 | 1.34-2.40 | 2.43E-07 | 2.22 |
| B*39 | 0.028 | 0.020 | 2.95E-02 | 1.40 | 1.03-1.89 | 1.25E-03 | 1.68 |
| B*40 | 0.045 | 0.064 | 3.05E-07 | 0.58 | 0.48-0.72 | 1.06E-03 | 0.70 |
| B*41 | 0.005 | 0.015 | 8.54E-03 | 0.48 | 0.28-0.83 | 1.01E-01 | 0.62 |
| B*44 | 0.105 | 0.139 | 6.22E-06 | 0.72 | 0.62-0.83 | 9.31E-02 | 0.88 |
| B*49 | 0.011 | 0.018 | 7.87E-02 | 0.69 | 0.46-1.04 | 4.63E-01 | 0.85 |
| B*50 | 0.015 | 0.011 | 1.48E-01 | 1.36 | 0.90-2.04 | 8.80E-04 | 0.49 |
| B*51 | 0.040 | 0.054 | 8.39E-02 | 0.82 | 0.66-1.03 | 9.29E-01 | 1.01 |
| B*55 | 0.023 | 0.016 | 1.06E-01 | 1.31 | 0.95-1.80 | 6.79E-03 | 1.58 |
| B*57 | 0.126 | 0.039 | 1.33E-42 | 3.66 | 3.04-4.41 | 7.25E-04 | 1.49 |
| C*01 | 0.035 | 0.034 | 8.64E-01 | 1.02 | 0.80-1.31 | 2.65E-01 | 1.16 |
| C*02 | 0.039 | 0.042 | 7.71E-01 | 0.97 | 0.76-1.22 | 1.70E-01 | 1.19 |
| C*03 | 0.111 | 0.120 | 2.11E-04 | 0.77 | 0.66-0.88 | 3.27E-01 | 0.93 |
| C*04 | 0.074 | 0.120 | 2.18E-08 | 0.63 | 0.54-0.74 | 2.08E-03 | 0.77 |
| C*05 | 0.067 | 0.081 | 6.76E-03 | 0.78 | 0.66-0.94 | 4.46E-01 | 0.93 |
| C*06 | 0.253 | 0.098 | 2.76E-77 | 3.57 | 3.12-4.09 | NA | NA |
| C*07 | 0.248 | 0.304 | 4.51E-09 | 0.74 | 0.67-0.82 | 8.60E-01 | 1.01 |
| C*08 | 0.036 | 0.039 | 7.50E-01 | 1.04 | 0.82-1.33 | 7.92E-02 | 1.25 |
| C*12 | 0.076 | 0.070 | 5.47E-02 | 1.19 | 1.00-1.43 | 4.99E-05 | 1.47 |
| C*14 | 0.013 | 0.011 | 1.73E-01 | 1.34 | 0.88-2.04 | 2.42E-02 | 1.65 |
| C*15 | 0.017 | 0.028 | 7.49E-03 | 0.64 | 0.47-0.89 | 2.26E-01 | 0.81 |
| C*16 | 0.026 | 0.040 | 1.82E-03 | 0.65 | 0.50-0.85 | 6.59E-02 | 0.77 |
| C*17 | 0.006 | 0.014 | 8.54E-03 | 0.49 | 0.28-0.83 | 8.48E-02 | 0.61 |
| DQA1*01 | 0.333 | 0.387 | 9.88E-06 | 0.79 | 0.71-0.88 | 1.24E-01 | 0.92 |
| DQA1*02 | 0.250 | 0.147 | 1.91E-25 | 2.01 | 1.76-2.29 | 1.20E-02 | 1.22 |
| DQA1*03 | 0.163 | 0.164 | 3.32E-01 | 0.94 | 0.82-1.07 | 4.75E-01 | 1.05 |
| DQA1*04 | 0.020 | 0.027 | 1.76E-01 | 0.79 | 0.56-1.11 | 1.94E-01 | 0.78 |
| DQA1*05 | 0.215 | 0.251 | 2.48E-03 | 0.83 | 0.74-0.94 | 8.60E-01 | 0.99 |
| DQB1*02 | 0.197 | 0.189 | 3.31E-01 | 1.07 | 0.94-1.21 | 8.91E-01 | 1.01 |
| DQB1*03 | 0.412 | 0.356 | 6.43E-06 | 1.27 | 1.15-1.41 | 1.93E-02 | 1.14 |
| DQB1*04 | 0.027 | 0.034 | 1.09E-01 | 0.78 | 0.58-1.06 | 1.11E-01 | 0.77 |
| DQB1*05 | 0.160 | 0.176 | 9.29E-02 | 0.89 | 0.78-1.02 | 4.67E-01 | 0.95 |
| DQB1*06 | 0.204 | 0.245 | 8.11E-05 | 0.79 | 0.70-0.89 | 1.34E-01 | 0.91 |
| DRB1*01 | 0.103 | 0.114 | 1.65E-01 | 0.89 | 0.75-1.05 | 7.34E-01 | 0.97 |
| DRB1*03 | 0.107 | 0.123 | 4.97E-02 | 0.85 | 0.72-1.00 | 5.26E-01 | 1.06 |
| DRB1*04 | 0.152 | 0.148 | 7.72E-01 | 0.98 | 0.85-1.13 | 2.95E-01 | 1.09 |
| DRB1*07 | 0.246 | 0.149 | 1.98E-20 | 1.90 | 1.66-2.18 | 1.99E-01 | 1.11 |
| DRB1*08 | 0.025 | 0.036 | 4.16E-02 | 0.72 | 0.53-0.99 | 9.06E-02 | 0.75 |
| DRB1*11 | 0.067 | 0.080 | 3.15E-01 | 0.90 | 0.73-1.11 | 5.08E-01 | 0.93 |
| DRB1*12 | 0.012 | 0.022 | 1.50E-03 | 0.50 | 0.32-0.76 | 3.97E-02 | 0.62 |
| DRB1*13 | 0.099 | 0.126 | 2.38E-04 | 0.73 | 0.61-0.86 | 2.08E-02 | 0.81 |
| DRB1*14 | 0.030 | 0.030 | 9.06E-01 | 0.98 | 0.72-1.34 | 5.86E-01 | 1.10 |
| DRB1*15 | 0.123 | 0.141 | 5.27E-02 | 0.86 | 0.74-1.00 | 7.28E-01 | 1.03 |
| DRB1*16 | 0.019 | 0.012 | 1.33E-03 | 2.09 | 1.33-3.29 | 2.85E-04 | 2.42 |
