# Supplementary material for: Psoriasis Patients Are Enriched for Genetic Variants That Protect against HIV-1 Disease
Source: PLoS Genet. 2012 Feb 16;8(2):e1002514. doi: 10.1371/journal.pgen.1002514 (PMC3343879; doi:10.1371/journal.pgen.1002514)
Supplement: Table S4 — Association results for the imputed amino acid residues in each of the classical HLA loci. P values and ORs were adjusted for ancestry, gender, and cohort. Only residues with frequency greater than 1% in the control group were analyzed. (DOC) [file pgen.1002514.s004.doc]

**Table S4. Association results for the imputed amino acid residues in each of the classical HLA loci.** P values and ORs were adjusted for ancestry, gender, and cohort. Only residues with frequency greater than 1% in the control group were analyzed.

| Locus | Amino acid position | A1 | A2 | Frequency in cases | Frequency in controls | OR | 95% CI | P-value | Omnibus P-value |
| --- | --- | --- | --- | --- | --- | --- | --- | --- | --- |
| A | -15 | V | L | 0.492 | 0.488 | 1.02 | 0.93-1.12 | 7.13E-01 |  |
| A | -11 | L | S | 0.090 | 0.117 | 0.71 | 0.61-0.83 | 1.84E-05 |  |
| A | 9 | F | - | 0.656 | 0.620 | 1.24 | 1.12-1.37 | 5.05E-05 | 1.32E-04 |
| A | 9 | S | - | 0.121 | 0.124 | 0.96 | 0.83-1.11 | 5.75E-01 |
| A | 9 | T | - | 0.063 | 0.080 | 0.74 | 0.61-0.90 | 2.17E-03 |
| A | 9 | Y | - | 0.160 | 0.176 | 0.85 | 0.74-0.96 | 1.15E-02 |
| A | 17 | S | R | 0.034 | 0.029 | 1.47 | 1.12-1.93 | 6.13E-03 |  |
| A | 43 | R | Q | 0.011 | 0.012 | 1.16 | 0.74-1.82 | 5.14E-01 |  |
| A | 44 | K | R | 0.216 | 0.165 | 1.42 | 1.26-1.59 | 9.53E-09 |  |
| A | 56 | R | G | 0.063 | 0.060 | 1.01 | 0.84-1.23 | 8.92E-01 |  |
| A | 62 | E | - | 0.088 | 0.116 | 0.81 | 0.69-0.95 | 8.98E-03 | 8.47E-05 |
| A | 62 | G | - | 0.312 | 0.271 | 1.21 | 1.09-1.34 | 2.30E-04 |
| A | 62 | L | - | 0.032 | 0.037 | 0.86 | 0.66-1.11 | 2.36E-01 |
| A | 62 | Q | - | 0.475 | 0.467 | 1.01 | 0.92-1.11 | 7.93E-01 |
| A | 62 | R | - | 0.093 | 0.110 | 0.81 | 0.69-0.95 | 8.30E-03 |
| A | 63 | E | - | 0.875 | 0.854 | 1.23 | 1.07-1.41 | 3.08E-03 | 1.14E-02 |
| A | 63 | N | - | 0.093 | 0.110 | 0.81 | 0.69-0.95 | 8.30E-03 |
| A | 63 | Q | - | 0.032 | 0.037 | 0.86 | 0.66-1.11 | 2.36E-01 |
| A | 65 | G | R | 0.088 | 0.116 | 0.81 | 0.69-0.95 | 8.98E-03 |
| A | 66 | K | N | 0.400 | 0.387 | 1.09 | 0.99-1.20 | 6.72E-02 |  |
| A | 67 | M | V | 0.216 | 0.165 | 1.42 | 1.26-1.59 | 9.53E-09 |  |
| A | 70 | Q | H | 0.269 | 0.303 | 0.83 | 0.75-0.92 | 4.13E-04 |  |
| A | 73 | I | T | 0.031 | 0.040 | 0.72 | 0.56-0.93 | 1.09E-02 |  |
| A | 74 | H | D | 0.312 | 0.271 | 1.21 | 1.09-1.34 | 2.35E-04 |  |
| A | 76 | A | - | 0.285 | 0.235 | 1.29 | 1.15-1.43 | 4.79E-06 | 2.09E-08 |
| A | 76 | E | - | 0.136 | 0.188 | 0.73 | 0.64-0.83 | 1.99E-06 |
| A | 76 | V | - | 0.579 | 0.577 | 0.98 | 0.89-1.08 | 6.75E-01 |
| A | 77 | D | - | 0.579 | 0.577 | 0.98 | 0.89-1.08 | 6.75E-01 | 3.30E-04 |
| A | 77 | N | - | 0.379 | 0.363 | 1.11 | 1.01-1.22 | 3.22E-02 |
| A | 77 | S | - | 0.041 | 0.059 | 0.67 | 0.54-0.83 | 2.64E-04 |
| A | 79 | R | G | 0.129 | 0.175 | 0.74 | 0.65-0.84 | 8.26E-06 |  |
| A | 80 | I | T | 0.129 | 0.175 | 0.74 | 0.65-0.84 | 8.26E-06 |  |
| A | 81 | A | L | 0.129 | 0.175 | 0.74 | 0.65-0.84 | 8.26E-06 |  |
| A | 82 | L | R | 0.129 | 0.175 | 0.74 | 0.65-0.84 | 8.26E-06 |  |
| A | 83 | R | G | 0.129 | 0.175 | 0.74 | 0.65-0.84 | 8.26E-06 |  |
| A | 90 | D | A | 0.325 | 0.288 | 1.19 | 1.07-1.31 | 8.25E-04 |  |
| A | 95 | I | - | 0.600 | 0.613 | 0.91 | 0.83-1.00 | 6.10E-02 | 3.71E-04 |
| A | 95 | L | - | 0.099 | 0.128 | 0.84 | 0.72-0.97 | 2.20E-02 |
| A | 95 | V | - | 0.302 | 0.258 | 1.21 | 1.09-1.34 | 2.60E-04 |
| A | 97 | I | - | 0.419 | 0.396 | 1.12 | 1.02-1.24 | 1.64E-02 | 5.60E-09 |
| A | 97 | M | - | 0.206 | 0.267 | 0.71 | 0.63-0.79 | 1.24E-09 |
| A | 97 | R | - | 0.375 | 0.337 | 1.16 | 1.05-1.28 | 2.49E-03 |
| A | 99 | F | Y | 0.088 | 0.116 | 0.81 | 0.69-0.95 | 8.98E-03 |  |
| A | 105 | P | S | 0.359 | 0.337 | 1.11 | 1.01-1.22 | 3.99E-02 |  |
| A | 107 | W | G | 0.313 | 0.271 | 1.21 | 1.10-1.34 | 1.92E-04 |  |
| A | 109 | L | F | 0.026 | 0.040 | 0.63 | 0.49-0.82 | 6.61E-04 |  |
| A | 114 | E | - | 0.033 | 0.029 | 1.44 | 1.10-1.90 | 8.97E-03 | 1.30E-05 |
| A | 114 | H | - | 0.409 | 0.396 | 1.10 | 1.00-1.21 | 4.51E-02 |
| A | 114 | Q | - | 0.111 | 0.138 | 0.73 | 0.64-0.84 | 1.75E-05 |
| A | 114 | R | - | 0.447 | 0.438 | 1.00 | 0.91-1.10 | 9.36E-01 |
| A | 116 | D | - | 0.558 | 0.576 | 0.87 | 0.79-0.96 | 4.35E-03 | 1.96E-03 |
| A | 116 | H | - | 0.033 | 0.029 | 1.44 | 1.10-1.90 | 8.97E-03 |
| A | 116 | Y | - | 0.409 | 0.396 | 1.10 | 1.00-1.21 | 4.51E-02 |
| A | 127 | K | N | 0.437 | 0.430 | 1.05 | 0.96-1.15 | 3.06E-01 |  |
| A | 142 | T | I | 0.350 | 0.314 | 1.15 | 1.04-1.26 | 6.39E-03 |  |
| A | 144 | Q | K | 0.192 | 0.227 | 0.80 | 0.71-0.90 | 1.80E-04 |  |
| A | 145 | H | R | 0.350 | 0.314 | 1.15 | 1.04-1.26 | 6.39E-03 |  |
| A | 149 | T | A | 0.054 | 0.058 | 0.86 | 0.71-1.06 | 1.59E-01 |  |
| A | 150 | V | A | 0.216 | 0.165 | 1.42 | 1.26-1.59 | 9.53E-09 |  |
| A | 151 | R | H | 0.136 | 0.167 | 0.80 | 0.70-0.91 | 8.00E-04 |  |
| A | 152 | A | - | 0.270 | 0.230 | 1.27 | 1.14-1.42 | 1.21E-05 | 3.00E-07 |
| A | 152 | E | - | 0.169 | 0.194 | 0.80 | 0.71-0.90 | 2.22E-04 |
| A | 152 | V | - | 0.529 | 0.549 | 0.92 | 0.84-1.01 | 9.71E-02 |
| A | 152 | W | - | 0.026 | 0.016 | 2.05 | 1.47-2.86 | 2.22E-05 |
| A | 156 | L | - | 0.553 | 0.562 | 0.93 | 0.85-1.02 | 1.46E-01 | 3.78E-08 |
| A | 156 | Q | - | 0.128 | 0.158 | 0.84 | 0.73-0.96 | 1.25E-02 |
| A | 156 | R | - | 0.216 | 0.165 | 1.42 | 1.26-1.59 | 9.53E-09 |
| A | 156 | W | - | 0.103 | 0.115 | 0.85 | 0.74-0.99 | 3.94E-02 |
| A | 158 | V | A | 0.216 | 0.165 | 1.42 | 1.26-1.59 | 9.53E-09 |  |
| A | 161 | D | E | 0.115 | 0.136 | 0.79 | 0.68-0.91 | 8.11E-04 |  |
| A | 163 | R | T | 0.324 | 0.288 | 1.19 | 1.07-1.31 | 7.94E-04 |  |
| A | 166 | D | E | 0.304 | 0.280 | 1.18 | 1.07-1.30 | 1.42E-03 |  |
| A | 167 | G | W | 0.304 | 0.280 | 1.18 | 1.07-1.30 | 1.42E-03 |  |
| A | 184 | A | P | 0.463 | 0.449 | 1.01 | 0.92-1.11 | 8.30E-01 |  |
| A | 193 | A | P | 0.494 | 0.489 | 0.96 | 0.88-1.06 | 4.44E-01 |  |
| A | 194 | V | I | 0.494 | 0.489 | 0.96 | 0.88-1.06 | 4.44E-01 |  |
| A | 207 | S | G | 0.494 | 0.489 | 0.96 | 0.88-1.06 | 4.44E-01 |  |
| A | 245 | V | A | 0.037 | 0.043 | 0.79 | 0.63-1.00 | 4.64E-02 |  |
| A | 246 | S | A | 0.144 | 0.175 | 0.74 | 0.66-0.85 | 6.87E-06 |  |
| A | 253 | Q | E | 0.494 | 0.489 | 0.96 | 0.88-1.06 | 4.44E-01 |  |
| A | 276 | L | P | 0.419 | 0.395 | 1.12 | 1.02-1.24 | 1.65E-02 |  |
| A | 282 | V | I | 0.088 | 0.116 | 0.81 | 0.69-0.95 | 8.79E-03 |  |
| A | 283 | H | P | 0.015 | 0.024 | 0.68 | 0.47-0.97 | 3.33E-02 |  |
| A | 294 | F | L | 0.494 | 0.489 | 0.96 | 0.88-1.06 | 4.49E-01 |  |
| A | 297 | M | V | 0.026 | 0.040 | 0.63 | 0.49-0.82 | 6.78E-04 |  |
| A | 298 | F | I | 0.090 | 0.117 | 0.71 | 0.61-0.83 | 1.84E-05 |  |
| A | 299 | A | T | 0.144 | 0.175 | 0.75 | 0.66-0.85 | 7.05E-06 |  |
| A | 307 | R | M | 0.090 | 0.117 | 0.71 | 0.61-0.83 | 1.84E-05 |  |
| A | 311 | N | K | 0.088 | 0.116 | 0.81 | 0.69-0.95 | 8.79E-03 |  |
| A | 321 | T | S | 0.419 | 0.395 | 1.12 | 1.02-1.24 | 1.65E-02 |  |
| A | 334 | M | V | 0.144 | 0.175 | 0.75 | 0.66-0.85 | 7.05E-06 |  |
| B | -23 | L | R | 0.299 | 0.316 | 0.89 | 0.81-0.99 | 2.46E-02 |  |
| B | -21 | M | T | 0.299 | 0.316 | 0.89 | 0.81-0.99 | 2.46E-02 |  |
| B | -16 | L | V | 0.325 | 0.307 | 1.09 | 0.99-1.21 | 7.80E-02 |  |
| B | -11 | S | W | 0.434 | 0.484 | 0.76 | 0.70-0.84 | 1.10E-08 |  |
| B | -10 | A | G | 0.375 | 0.418 | 0.80 | 0.73-0.88 | 2.53E-06 |  |
| B | -8 | L | V | 0.463 | 0.506 | 0.79 | 0.72-0.86 | 2.54E-07 |  |
| B | 9 | D | - | 0.100 | 0.109 | 0.88 | 0.76-1.03 | 1.04E-01 | 1.03E-03 |
| B | 9 | H | - | 0.196 | 0.222 | 0.84 | 0.75-0.94 | 2.86E-03 |
| B | 9 | Y | - | 0.703 | 0.669 | 1.21 | 1.09-1.33 | 2.29E-04 |
| B | 11 | S | A | 0.287 | 0.296 | 0.92 | 0.83-1.02 | 1.14E-01 |  |
| B | 12 | V | M | 0.318 | 0.326 | 0.95 | 0.86-1.05 | 2.84E-01 |  |
| B | 24 | A | - | 0.324 | 0.290 | 1.23 | 1.11-1.36 | 5.18E-05 | 2.22E-04 |
| B | 24 | S | - | 0.365 | 0.392 | 0.88 | 0.80-0.97 | 7.60E-03 |
| B | 24 | T | - | 0.311 | 0.318 | 0.94 | 0.85-1.04 | 2.53E-01 |
| B | 30 | G | D | 0.036 | 0.056 | 0.65 | 0.51-0.81 | 2.05E-04 |  |
| B | 32 | L | Q | 0.248 | 0.292 | 0.76 | 0.69-0.85 | 5.95E-07 |  |
| B | 41 | T | A | 0.261 | 0.281 | 0.88 | 0.79-0.98 | 1.75E-02 |  |
| B | 45 | E | - | 0.381 | 0.381 | 0.97 | 0.89-1.07 | 5.80E-01 | 3.83E-40 |
| B | 45 | K | - | 0.198 | 0.255 | 0.69 | 0.61-0.77 | 8.39E-11 |
| B | 45 | M | - | 0.245 | 0.123 | 2.21 | 1.96-2.50 | 9.39E-37 |
| B | 45 | T | - | 0.177 | 0.241 | 0.76 | 0.67-0.85 | 2.95E-06 |
| B | 46 | A | E | 0.245 | 0.123 | 2.21 | 1.96-2.50 | 9.39E-37 |  |
| B | 62 | G | R | 0.135 | 0.048 | 3.20 | 2.69-3.81 | 2.03E-39 |  |
| B | 63 | E | N | 0.536 | 0.453 | 1.35 | 1.23-1.48 | 3.05E-10 |  |
| B | 65 | R | Q | 0.137 | 0.051 | 3.06 | 2.58-3.63 | 1.00E-37 |  |
| B | 66 | N | I | 0.137 | 0.051 | 3.06 | 2.58-3.63 | 1.00E-37 |  |
| B | 67 | C | - | 0.154 | 0.124 | 1.36 | 1.19-1.56 | 6.47E-06 | 4.00E-45 |
| B | 67 | F | - | 0.199 | 0.259 | 0.75 | 0.67-0.84 | 4.41E-07 |
| B | 67 | M | - | 0.137 | 0.051 | 3.06 | 2.58-3.63 | 1.00E-37 |
| B | 67 | S | - | 0.385 | 0.422 | 0.83 | 0.75-0.91 | 1.03E-04 |
| B | 67 | Y | - | 0.126 | 0.144 | 0.79 | 0.69-0.90 | 4.87E-04 |
| B | 69 | A | T | 0.313 | 0.232 | 1.44 | 1.30-1.59 | 8.14E-12 |  |
| B | 70 | K | - | 0.050 | 0.037 | 1.34 | 1.07-1.67 | 1.08E-02 | 1.35E-40 |
| B | 70 | N | - | 0.688 | 0.768 | 0.70 | 0.63-0.77 | 7.57E-12 |
| B | 70 | Q | - | 0.126 | 0.144 | 0.79 | 0.69-0.90 | 5.64E-04 |
| B | 70 | S | - | 0.137 | 0.051 | 3.06 | 2.58-3.63 | 9.33E-38 |
| B | 71 | A | T | 0.313 | 0.232 | 1.44 | 1.30-1.60 | 7.57E-12 |  |
| B | 74 | D | Y | 0.340 | 0.348 | 0.92 | 0.84-1.01 | 8.65E-02 |  |
| B | 77 | D | - | 0.084 | 0.051 | 1.64 | 1.37-1.97 | 1.02E-07 | 6.17E-26 |
| B | 77 | N | - | 0.402 | 0.327 | 1.49 | 1.35-1.64 | 1.13E-15 |
| B | 77 | S | - | 0.514 | 0.623 | 0.60 | 0.55-0.63 | 2.00E-25 |
| B | 80 | I | - | 0.233 | 0.159 | 1.80 | 1.60-2.03 | 8.28E-22 | 1.76E-29 |
| B | 80 | N | - | 0.514 | 0.623 | 0.60 | 0.55-0.66 | 1.28E-25 |
| B | 80 | T | - | 0.253 | 0.218 | 1.22 | 1.09-1.36 | 3.41E-04 |
| B | 81 | A | L | 0.402 | 0.327 | 1.49 | 1.35-1.64 | 1.13E-15 |  |
| B | 82 | L | R | 0.486 | 0.377 | 1.66 | 1.51-1.83 | 1.28E-25 |  |
| B | 83 | R | G | 0.486 | 0.377 | 1.66 | 1.51-1.83 | 1.28E-25 |  |
| B | 94 | I | T | 0.298 | 0.284 | 1.11 | 1.01-1.23 | 3.28E-02 |  |
| B | 95 | I | - | 0.325 | 0.298 | 1.18 | 1.07-1.30 | 9.21E-04 | 4.25E-08 |
| B | 95 | L | - | 0.495 | 0.539 | 0.76 | 0.70-0.84 | 1.14E-08 |
| B | 95 | W | - | 0.180 | 0.163 | 1.25 | 1.11-1.41 | 3.79E-04 |
| B | 97 | N | - | 0.050 | 0.037 | 1.34 | 1.08-1.68 | 9.43E-03 | 1.58E-53 |
| B | 97 | R | - | 0.434 | 0.513 | 0.73 | 0.66-0.80 | 1.56E-11 |
| B | 97 | S | - | 0.208 | 0.251 | 0.73 | 0.66-0.82 | 2.71E-08 |
| B | 97 | T | - | 0.144 | 0.119 | 1.36 | 1.19-1.56 | 9.75E-06 |
| B | 97 | V | - | 0.126 | 0.039 | 3.66 | 3.04-4.41 | 1.21E-42 |
| B | 97 | W | - | 0.038 | 0.041 | 1.04 | 0.81-1.33 | 7.68E-01 |
| B | 99 | S | Y | 0.027 | 0.014 | 1.95 | 1.41-2.71 | 6.14E-05 |  |
| B | 103 | L | V | 0.192 | 0.189 | 1.11 | 0.99-1.26 | 7.39E-02 |  |
| B | 109 | F | L | 0.005 | 0.014 | 0.55 | 0.31-0.98 | 4.08E-02 |  |
| B | 113 | Y | H | 0.263 | 0.270 | 0.98 | 0.89-1.09 | 7.42E-01 |  |
| B | 114 | D | - | 0.484 | 0.513 | 0.85 | 0.78-0.94 | 7.67E-04 | 5.77E-05 |
| B | 114 | H | - | 0.060 | 0.041 | 1.47 | 1.19-1.81 | 2.96E-04 |
| B | 114 | N | - | 0.456 | 0.446 | 1.09 | 0.99-1.20 | 6.96E-02 |
| B | 116 | D | - | 0.164 | 0.179 | 0.89 | 0.78-1.00 | 5.33E-02 | 1.80E-23 |
| B | 116 | F | - | 0.131 | 0.113 | 1.33 | 1.15-1.53 | 1.08E-04 |
| B | 116 | L | - | 0.125 | 0.082 | 1.65 | 1.42-1.92 | 9.92E-11 |
| B | 116 | S | - | 0.277 | 0.234 | 1.22 | 1.10-1.36 | 1.93E-04 |
| B | 116 | Y | - | 0.303 | 0.392 | 0.66 | 0.60-0.73 | 7.96E-17 |
| B | 131 | R | S | 0.249 | 0.311 | 0.67 | 0.60-0.74 | 3.10E-14 |  |
| B | 143 | S | T | 0.038 | 0.052 | 0.60 | 0.48-0.75 | 8.00E-06 |  |
| B | 145 | L | R | 0.063 | 0.025 | 2.78 | 2.19-3.53 | 4.70E-17 |  |
| B | 147 | L | W | 0.038 | 0.052 | 0.60 | 0.48-0.75 | 8.00E-06 |  |
| B | 152 | E | V | 0.291 | 0.339 | 0.78 | 0.71-0.87 | 1.90E-06 |  |
| B | 156 | D | - | 0.213 | 0.235 | 0.88 | 0.78-0.98 | 1.88E-02 | 1.16E-10 |
| B | 156 | L | - | 0.633 | 0.574 | 1.36 | 1.24-1.50 | 1.39E-10 |
| B | 156 | R | - | 0.101 | 0.135 | 0.69 | 0.59-0.79 | 3.65E-07 |
| B | 156 | W | - | 0.053 | 0.056 | 0.80 | 0.65-0.98 | 3.07E-02 |
| B | 158 | T | A | 0.063 | 0.043 | 1.62 | 1.30-2.00 | 1.16E-05 |  |
| B | 163 | E | - | 0.265 | 0.251 | 0.97 | 0.88-1.08 | 5.70E-01 | 3.30E-01 |
| B | 163 | L | - | 0.444 | 0.455 | 1.01 | 0.92-1.11 | 8.58E-01 |
| B | 163 | T | - | 0.291 | 0.295 | 1.02 | 0.92-1.13 | 1.05E-01 |
| B | 167 | S | W | 0.113 | 0.146 | 0.74 | 0.64-0.85 | 2.08E-05 |  |
| B | 171 | H | Y | 0.120 | 0.162 | 0.78 | 0.68-0.90 | 4.49E-04 |  |
| B | 177 | D | E | 0.241 | 0.298 | 0.68 | 0.61-0.76 | 8.92E-13 |  |
| B | 178 | K | T | 0.135 | 0.174 | 0.65 | 0.57-0.74 | 3.49E-11 |  |
| B | 180 | E | Q | 0.242 | 0.298 | 0.68 | 0.62-0.76 | 1.13E-12 |  |
| B | 194 | V | I | 0.114 | 0.171 | 0.71 | 0.62-0.82 | 1.24E-06 |  |
| B | 199 | V | A | 0.106 | 0.140 | 0.72 | 0.62-0.83 | 6.87E-06 |  |
| B | 282 | I | V | 0.297 | 0.365 | 0.78 | 0.71-0.87 | 1.81E-06 |  |
| B | 305 | T | A | 0.297 | 0.365 | 0.78 | 0.71-0.87 | 1.81E-06 |  |
| B | 325 | S | C | 0.398 | 0.449 | 0.88 | 0.80-0.97 | 9.31E-03 |  |
| C | -17 | Q | R | 0.006 | 0.014 | 0.54 | 0.32-0.90 | 1.88E-02 |  |
| C | -16 | A | T | 0.253 | 0.318 | 0.75 | 0.68-0.83 | 3.06E-09 |  |
| C | -14 | L | I | 0.309 | 0.386 | 0.75 | 0.68-0.82 | 1.14E-10 |  |
| C | -8 | G | A | 0.247 | 0.304 | 0.77 | 0.70-0.84 | 4.66E-08 |  |
| C | -4 | I | T | 0.006 | 0.014 | 0.54 | 0.32-0.90 | 1.88E-02 |  |
| C | 1 | G | C | 0.190 | 0.254 | 0.69 | 0.62-0.77 | 5.21E-12 |  |
| C | 6 | K | R | 0.036 | 0.034 | 1.03 | 0.82-1.29 | 8.24E-01 |  |
| C | 9 | D | - | 0.501 | 0.401 | 1.43 | 1.31-1.56 | 1.26E-15 | 2.43E-15 |
| C | 9 | F | - | 0.036 | 0.034 | 1.03 | 0.82-1.29 | 8.24E-01 |
| C | 9 | S | - | 0.087 | 0.132 | 0.72 | 0.62-0.83 | 5.47E-06 |
| C | 9 | Y | - | 0.377 | 0.433 | 0.79 | 0.73-0.87 | 3.18E-07 |
| C | 11 | S | A | 0.123 | 0.166 | 0.78 | 0.69-0.88 | 6.94E-05 |  |
| C | 14 | W | R | 0.074 | 0.121 | 0.67 | 0.58-0.78 | 2.46E-07 |  |
| C | 16 | S | G | 0.039 | 0.041 | 0.97 | 0.78-1.21 | 8.06E-01 |  |
| C | 21 | H | R | 0.167 | 0.188 | 0.80 | 0.71-0.89 | 8.03E-05 |  |
| C | 24 | S | A | 0.536 | 0.436 | 1.42 | 1.31-1.55 | 1.51E-15 |  |
| C | 35 | Q | R | 0.103 | 0.121 | 0.87 | 0.76-1.00 | 5.15E-02 |  |
| C | 49 | E | A | 0.074 | 0.121 | 0.67 | 0.58-0.78 | 2.46E-07 |  |
| C | 66 | N | K | 0.141 | 0.187 | 0.79 | 0.70-0.88 | 5.35E-05 |  |
| C | 73 | T | A | 0.344 | 0.394 | 0.79 | 0.72-0.86 | 1.55E-07 |  |
| C | 77 | N | S | 0.457 | 0.386 | 1.36 | 1.25-1.48 | 2.69E-12 |  |
| C | 80 | K | N | 0.457 | 0.386 | 1.36 | 1.25-1.48 | 2.69E-12 |  |
| C | 90 | A | D | 0.426 | 0.478 | 0.81 | 0.74-0.88 | 2.32E-06 |  |
| C | 91 | R | G | 0.054 | 0.050 | 0.96 | 0.79-1.17 | 6.94E-01 |  |
| C | 94 | I | T | 0.128 | 0.146 | 0.76 | 0.67-0.86 | 2.07E-05 |  |
| C | 95 | F | - | 0.013 | 0.016 | 0.82 | 0.57-1.17 | 2.68E-01 | 1.60E-06 |
| C | 95 | I | - | 0.132 | 0.158 | 0.74 | 0.65-0.83 | 1.22E-06 |
| C | 95 | L | - | 0.854 | 0.826 | 1.36 | 1.21-1.53 | 4.38E-07 |
| C | 97 | W | R | 0.397 | 0.244 | 1.95 | 1.78-2.14 | 4.56E-45 |  |
| C | 99 | C | - | 0.036 | 0.034 | 1.03 | 0.82-1.29 | 8.24E-01 | 4.29E-09 |
| C | 99 | F | - | 0.087 | 0.132 | 0.72 | 0.62-0.83 | 5.47E-06 |
| C | 99 | S | - | 0.110 | 0.129 | 0.78 | 0.68-0.89 | 2.17E-04 |
| C | 99 | Y | - | 0.768 | 0.706 | 1.35 | 1.22-1.49 | 3.02E-09 |
| C | 103 | V | L | 0.111 | 0.119 | 0.79 | 0.69-0.90 | 5.10E-04 |  |
| C | 113 | H | Y | 0.016 | 0.027 | 0.67 | 0.49-0.91 | 1.09E-02 |  |
| C | 114 | N | D | 0.182 | 0.256 | 0.73 | 0.65-0.81 | 5.55E-09 |  |
| C | 116 | F | - | 0.197 | 0.274 | 0.73 | 0.65-0.80 | 9.99E-10 | 1.64E-15 |
| C | 116 | L | - | 0.015 | 0.024 | 0.68 | 0.49-0.95 | 2.28E-02 |
| C | 116 | S | - | 0.642 | 0.550 | 1.45 | 1.33-1.58 | 2.27E-16 |
| C | 116 | Y | - | 0.146 | 0.152 | 0.83 | 0.73-0.93 | 2.18E-03 |
| C | 138 | K | T | 0.103 | 0.121 | 0.87 | 0.76-1.00 | 4.56E-02 |  |
| C | 143 | S | T | 0.006 | 0.014 | 0.54 | 0.32-0.90 | 1.88E-02 |  |
| C | 147 | L | W | 0.253 | 0.318 | 0.75 | 0.68-0.83 | 3.06E-09 |  |
| C | 152 | A | E | 0.273 | 0.344 | 0.74 | 0.67-0.81 | 1.32E-10 |  |
| C | 156 | D | - | 0.013 | 0.016 | 0.82 | 0.57-1.17 | 2.68E-01 | 3.89E-51 |
| C | 156 | L | - | 0.367 | 0.448 | 0.69 | 0.63-0.76 | 2.84E-16 |
| C | 156 | Q | - | 0.026 | 0.041 | 0.67 | 0.52-0.87 | 2.44E-03 |
| C | 156 | R | - | 0.225 | 0.287 | 0.79 | 0.71-0.87 | 2.83E-06 |
| C | 156 | W | - | 0.369 | 0.209 | 2.12 | 1.93-2.33 | 1.02E-52 |
| C | 163 | E | - | 0.045 | 0.055 | 0.87 | 0.72-1.07 | 1.88E-01 | 5.15E-04 |
| C | 163 | L | - | 0.111 | 0.119 | 0.79 | 0.69-0.90 | 5.10E-04 |
| C | 163 | T | - | 0.845 | 0.826 | 1.25 | 1.11-1.41 | 1.63E-04 |
| C | 170 | G | R | 0.006 | 0.014 | 0.54 | 0.32-0.90 | 1.88E-02 |  |
| C | 173 | K | E | 0.111 | 0.119 | 0.79 | 0.69-0.90 | 5.10E-04 |  |
| C | 177 | K | E | 0.116 | 0.137 | 0.86 | 0.76-0.98 | 2.55E-02 |  |
| C | 184 | H | - | 0.747 | 0.683 | 1.33 | 1.21-1.47 | 3.06E-09 | 3.13E-09 |
| C | 184 | P | - | 0.247 | 0.304 | 0.77 | 0.70-0.84 | 4.66E-08 |
| C | 184 | R | - | 0.006 | 0.014 | 0.54 | 0.32-0.90 | 1.88E-02 |
| C | 193 | L | P | 0.026 | 0.041 | 0.68 | 0.53-0.88 | 3.45E-03 |  |
| C | 194 | L | V | 0.247 | 0.304 | 0.77 | 0.70-0.84 | 4.66E-08 |  |
| C | 211 | T | A | 0.039 | 0.041 | 0.97 | 0.78-1.21 | 8.06E-01 |  |
| C | 219 | W | R | 0.233 | 0.285 | 0.75 | 0.68-0.83 | 1.17E-08 |  |
| C | 248 | M | V | 0.036 | 0.034 | 1.03 | 0.82-1.29 | 8.24E-01 |  |
| C | 253 | Q | E | 0.253 | 0.318 | 0.75 | 0.68-0.83 | 3.06E-09 |  |
| C | 261 | M | V | 0.247 | 0.304 | 0.77 | 0.70-0.84 | 4.66E-08 |  |
| C | 267 | Q | P | 0.253 | 0.318 | 0.75 | 0.68-0.83 | 3.06E-09 |  |
| C | 270 | C | L | 0.006 | 0.014 | 0.54 | 0.32-0.90 | 1.88E-02 |  |
| C | 273 | S | R | 0.247 | 0.304 | 0.77 | 0.70-0.84 | 4.66E-08 |  |
| C | 275 | E | - | 0.818 | 0.745 | 1.37 | 1.23-1.53 | 5.55E-09 | 6.10E-10 |
| C | 275 | G | - | 0.103 | 0.121 | 0.87 | 0.76-1.00 | 5.15E-02 |
| C | 275 | K | - | 0.079 | 0.135 | 0.65 | 0.57-0.76 | 1.38E-08 |
| C | 284 | N | I | 0.006 | 0.014 | 0.54 | 0.32-0.90 | 1.88E-02 |  |
| C | 285 | L | - | 0.006 | 0.014 | 0.54 | 0.32-0.90 | 1.88E-02 | 3.13E-09 |
| C | 285 | M | - | 0.247 | 0.304 | 0.77 | 0.70-0.84 | 4.66E-08 |
| C | 285 | V | - | 0.747 | 0.683 | 1.33 | 1.21-1.47 | 3.06E-09 |
| C | 289 | S | A | 0.006 | 0.014 | 0.54 | 0.32-0.90 | 1.88E-02 |  |
| C | 291 | P | L | 0.006 | 0.014 | 0.54 | 0.32-0.90 | 1.88E-02 |  |
| C | 295 | V | A | 0.247 | 0.304 | 0.77 | 0.70-0.84 | 4.66E-08 |  |
| C | 303 | M | V | 0.074 | 0.121 | 0.67 | 0.58-0.78 | 2.46E-07 |  |
| C | 304 | M | V | 0.449 | 0.316 | 1.73 | 1.58-1.89 | 1.35E-33 |  |
| C | 305 | T | A | 0.247 | 0.304 | 0.77 | 0.70-0.84 | 4.66E-08 |  |
| C | 306 | A | V | 0.253 | 0.318 | 0.75 | 0.68-0.83 | 3.06E-09 |  |
| C | 307 | M | V | 0.247 | 0.304 | 0.77 | 0.70-0.84 | 4.66E-08 |  |
| C | 308 | I | M | 0.006 | 0.014 | 0.54 | 0.32-0.90 | 1.88E-02 |  |
| C | 309 | H | C | 0.006 | 0.014 | 0.54 | 0.32-0.90 | 1.88E-02 |  |
| C | 326 | C | S | 0.247 | 0.304 | 0.77 | 0.70-0.84 | 4.66E-08 |  |
| C | 339 | T | A | 0.247 | 0.304 | 0.77 | 0.70-0.84 | 4.66E-08 |  |
| DQA1 | -15 | L | M | 0.359 | 0.426 | 0.75 | 0.67-0.83 | 1.15E-07 |  |
| DQA1 | 11 | C | Y | 0.339 | 0.399 | 0.77 | 0.69-0.85 | 1.35E-06 |  |
| DQA1 | 18 | F | S | 0.339 | 0.399 | 0.77 | 0.69-0.85 | 1.35E-06 |  |
| DQA1 | 25 | F | Y | 0.315 | 0.214 | 1.71 | 1.52-1.93 | 1.21E-18 |  |
| DQA1 | 26 | S | T | 0.166 | 0.166 | 0.94 | 0.82-1.08 | 4.01E-01 |  |
| DQA1 | 34 | E | Q | 0.537 | 0.456 | 1.36 | 1.23-1.51 | 4.91E-09 |  |
| DQA1 | 40 | G | E | 0.239 | 0.284 | 0.81 | 0.72-0.91 | 4.90E-04 |  |
| DQA1 | 41 | K | R | 0.060 | 0.062 | 0.90 | 0.72-1.12 | 3.31E-01 |  |
| DQA1 | 45 | A | V | 0.339 | 0.399 | 0.77 | 0.69-0.85 | 1.35E-06 |  |
| DQA1 | 47 | C | - | 0.239 | 0.284 | 0.81 | 0.72-0.91 | 4.90E-04 | 1.17E-23 |
| DQA1 | 47 | K | - | 0.256 | 0.152 | 1.99 | 1.74-2.27 | 3.40E-24 |
| DQA1 | 47 | Q | - | 0.166 | 0.166 | 0.94 | 0.82-1.08 | 4.01E-01 |
| DQA1 | 47 | R | - | 0.339 | 0.399 | 0.77 | 0.69-0.85 | 1.35E-06 |
| DQA1 | 48 | W | L | 0.339 | 0.399 | 0.77 | 0.69-0.85 | 1.35E-06 |  |
| DQA1 | 50 | E | - | 0.339 | 0.399 | 0.77 | 0.69-0.85 | 1.35E-06 | 2.26E-14 |
| DQA1 | 50 | L | - | 0.422 | 0.317 | 1.54 | 1.38-1.71 | 4.25E-15 |
| DQA1 | 50 | V | - | 0.239 | 0.284 | 0.81 | 0.72-0.91 | 4.90E-04 |
| DQA1 | 51 | L | F | 0.239 | 0.284 | 0.81 | 0.72-0.91 | 4.90E-04 |  |
| DQA1 | 52 | H | - | 0.256 | 0.152 | 1.99 | 1.74-2.27 | 3.40E-24 | 2.70E-24 |
| DQA1 | 52 | R | - | 0.406 | 0.450 | 0.82 | 0.74-0.91 | 1.96E-04 |
| DQA1 | 52 | S | - | 0.339 | 0.399 | 0.77 | 0.69-0.85 | 1.35E-06 |
| DQA1 | 53 | K | - | 0.339 | 0.399 | 0.77 | 0.69-0.85 | 1.35E-06 | 2.26E-14 |
| DQA1 | 53 | Q | - | 0.239 | 0.284 | 0.81 | 0.72-0.91 | 4.90E-04 |
| DQA1 | 53 | R | - | 0.422 | 0.317 | 1.54 | 1.38-1.71 | 4.25E-15 |
| DQA1 | 54 | L | F | 0.256 | 0.152 | 1.99 | 1.74-2.27 | 3.40E-24 |  |
| DQA1 | 55 | G | R | 0.339 | 0.399 | 0.77 | 0.69-0.85 | 1.35E-06 |  |
| DQA1 | 61 | G | F | 0.339 | 0.399 | 0.77 | 0.69-0.85 | 1.35E-06 |  |
| DQA1 | 64 | R | T | 0.339 | 0.399 | 0.77 | 0.69-0.85 | 1.35E-06 |  |
| DQA1 | 66 | M | I | 0.339 | 0.399 | 0.77 | 0.69-0.85 | 1.35E-06 |  |
| DQA1 | 69 | A | - | 0.339 | 0.399 | 0.77 | 0.69-0.85 | 1.35E-06 | 5.27E-07 |
| DQA1 | 69 | L | - | 0.642 | 0.574 | 1.33 | 1.20-1.48 | 1.15E-07 |
| DQA1 | 69 | T | - | 0.020 | 0.028 | 0.74 | 0.53-1.05 | 9.48E-02 |
| DQA1 | 75 | S | I | 0.220 | 0.257 | 0.83 | 0.73-0.94 | 2.72E-03 |  |
| DQA1 | 76 | L | - | 0.495 | 0.436 | 1.33 | 1.20-1.48 | 9.41E-08 | 1.83E-07 |
| DQA1 | 76 | M | - | 0.339 | 0.399 | 0.77 | 0.69-0.85 | 1.35E-06 |
| DQA1 | 76 | V | - | 0.166 | 0.166 | 0.94 | 0.82-1.08 | 4.01E-01 |
| DQA1 | 80 | Y | S | 0.339 | 0.399 | 0.77 | 0.69-0.85 | 1.35E-06 |  |
| DQA1 | 107 | I | T | 0.220 | 0.257 | 0.83 | 0.73-0.94 | 2.72E-03 |  |
| DQA1 | 129 | Q | H | 0.279 | 0.336 | 0.77 | 0.69-0.86 | 4.92E-06 |  |
| DQA1 | 130 | A | S | 0.060 | 0.062 | 0.90 | 0.72-1.12 | 3.31E-01 |  |
| DQA1 | 156 | L | F | 0.220 | 0.257 | 0.83 | 0.73-0.94 | 2.72E-03 |  |
| DQA1 | 161 | E | D | 0.220 | 0.257 | 0.83 | 0.73-0.94 | 2.72E-03 |  |
| DQA1 | 163 | S | I | 0.220 | 0.257 | 0.83 | 0.73-0.94 | 2.72E-03 |  |
| DQA1 | 175 | E | - | 0.442 | 0.345 | 1.48 | 1.33-1.64 | 4.53E-13 | 2.54E-12 |
| DQA1 | 175 | K | - | 0.220 | 0.257 | 0.83 | 0.73-0.94 | 2.72E-03 |
| DQA1 | 175 | Q | - | 0.339 | 0.399 | 0.77 | 0.69-0.85 | 1.35E-06 |
| DQA1 | 187 | T | A | 0.166 | 0.166 | 0.94 | 0.82-1.08 | 4.01E-01 |  |
| DQA1 | 207 | M | V | 0.164 | 0.197 | 0.81 | 0.71-0.93 | 2.26E-03 |  |
| DQA1 | 215 | L | F | 0.422 | 0.317 | 1.54 | 1.38-1.71 | 4.25E-15 |  |
| DQA1 | 218 | Q | R | 0.339 | 0.399 | 0.77 | 0.69-0.85 | 1.35E-06 |  |
| DQB1 | -27 | S | A | 0.140 | 0.156 | 0.89 | 0.75-1.05 | 1.80E-01 |  |
| DQB1 | -21 | D | G | 0.274 | 0.313 | 0.83 | 0.73-0.94 | 3.91E-03 |  |
| DQB1 | -18 | A | V | 0.436 | 0.473 | 0.89 | 0.79-1.00 | 4.83E-02 |  |
| DQB1 | -10 | S | A | 0.222 | 0.224 | 1.00 | 0.87-1.15 | 9.73E-01 |  |
| DQB1 | -9 | I | M | 0.140 | 0.156 | 0.89 | 0.75-1.05 | 1.80E-01 |  |
| DQB1 | -6 | S | T | 0.274 | 0.313 | 0.83 | 0.73-0.94 | 3.91E-03 |  |
| DQB1 | -5 | L | - | 0.134 | 0.157 | 0.82 | 0.70-0.97 | 2.07E-02 | 1.29E-02 |
| DQB1 | -5 | P | - | 0.726 | 0.687 | 1.21 | 1.06-1.38 | 3.91E-03 |
| DQB1 | -5 | S | - | 0.140 | 0.156 | 0.89 | 0.75-1.05 | 1.80E-01 |
| DQB1 | -4 | L | V | 0.274 | 0.313 | 0.83 | 0.73-0.94 | 3.91E-03 |  |
| DQB1 | 9 | F | Y | 0.142 | 0.166 | 0.84 | 0.73-0.96 | 1.21E-02 |  |
| DQB1 | 13 | A | G | 0.194 | 0.212 | 0.92 | 0.81-1.04 | 1.78E-01 |  |
| DQB1 | 14 | L | M | 0.160 | 0.176 | 0.89 | 0.78-1.02 | 9.29E-02 |  |
| DQB1 | 26 | G | - | 0.186 | 0.210 | 0.86 | 0.76-0.98 | 2.32E-02 | 1.05E-02 |
| DQB1 | 26 | L | - | 0.620 | 0.578 | 1.17 | 1.05-1.30 | 3.09E-03 |
| DQB1 | 26 | Y | - | 0.194 | 0.212 | 0.92 | 0.81-1.04 | 1.78E-01 |
| DQB1 | 28 | S | T | 0.198 | 0.189 | 1.07 | 0.94-1.21 | 3.15E-01 |  |
| DQB1 | 30 | H | - | 0.235 | 0.274 | 0.80 | 0.72-0.90 | 2.31E-04 | 1.08E-03 |
| DQB1 | 30 | S | - | 0.198 | 0.189 | 1.07 | 0.94-1.21 | 3.15E-01 |
| DQB1 | 30 | Y | - | 0.568 | 0.537 | 1.13 | 1.02-1.25 | 1.53E-02 |
| DQB1 | 37 | I | Y | 0.198 | 0.189 | 1.07 | 0.94-1.21 | 3.15E-01 |  |
| DQB1 | 38 | V | A | 0.364 | 0.369 | 0.99 | 0.89-1.09 | 7.77E-01 |  |
| DQB1 | 45 | E | G | 0.188 | 0.208 | 0.9 | 0.79-1.03 | 1.12E-01 |  |
| DQB1 | 46 | E | V | 0.198 | 0.189 | 1.07 | 0.94-1.21 | 3.15E-01 |  |
| DQB1 | 47 | F | Y | 0.198 | 0.189 | 1.07 | 0.94-1.21 | 3.15E-01 |  |
| DQB1 | 52 | L | P | 0.198 | 0.189 | 1.07 | 0.94-1.21 | 3.15E-01 |  |
| DQB1 | 53 | Q | L | 0.363 | 0.421 | 0.78 | 0.70-0.86 | 2.35E-06 |  |
| DQB1 | 55 | L | - | 0.198 | 0.189 | 1.07 | 0.94-1.21 | 3.15E-01 | 3.67E-07 |
| DQB1 | 55 | P | - | 0.412 | 0.356 | 1.27 | 1.15-1.41 | 6.43E-06 |
| DQB1 | 55 | R | - | 0.390 | 0.455 | 0.76 | 0.68-0.84 | 1.51E-07 |
| DQB1 | 56 | L | P | 0.027 | 0.034 | 0.78 | 0.58-1.06 | 1.09E-01 |  |
| DQB1 | 57 | A | - | 0.304 | 0.288 | 1.05 | 0.94-1.18 | 3.66E-01 | 2.07E-05 |
| DQB1 | 57 | D | - | 0.544 | 0.530 | 1.08 | 0.97-1.19 | 1.44E-01 |
| DQB1 | 57 | S | - | 0.023 | 0.017 | 1.73 | 1.18-2.53 | 5.22E-03 |
| DQB1 | 57 | V | - | 0.128 | 0.166 | 0.74 | 0.64-0.85 | 2.50E-05 |
| DQB1 | 66 | D | E | 0.231 | 0.228 | 1.03 | 0.91-1.16 | 6.71E-01 |  |
| DQB1 | 67 | I | V | 0.231 | 0.228 | 1.03 | 0.91-1.16 | 6.71E-01 |  |
| DQB1 | 70 | E | - | 0.027 | 0.035 | 0.76 | 0.57-1.03 | 7.51E-02 | 4.55E-04 |
| DQB1 | 70 | G | - | 0.331 | 0.369 | 0.84 | 0.76-0.94 | 1.41E-03 |
| DQB1 | 70 | R | - | 0.643 | 0.596 | 1.23 | 1.10-1.36 | 1.53E-04 |
| DQB1 | 71 | A | - | 0.159 | 0.175 | 0.9 | 0.78-1.03 | 1.11E-01 | 8.88E-02 |
| DQB1 | 71 | D | - | 0.027 | 0.035 | 0.76 | 0.57-1.03 | 7.51E-02 |
| DQB1 | 71 | K | - | 0.198 | 0.189 | 1.07 | 0.94-1.21 | 3.15E-01 |
| DQB1 | 71 | T | - | 0.616 | 0.601 | 1.06 | 0.95-1.17 | 3.04E-01 |
| DQB1 | 74 | A | - | 0.198 | 0.189 | 1.07 | 0.94-1.21 | 3.15E-01 | 6.69E-02 |
| DQB1 | 74 | E | - | 0.616 | 0.601 | 1.06 | 0.95-1.17 | 3.04E-01 |
| DQB1 | 74 | S | - | 0.186 | 0.210 | 0.86 | 0.76-0.98 | 2.32E-02 |
| DQB1 | 75 | V | L | 0.384 | 0.399 | 0.95 | 0.85-1.05 | 3.04E-01 |  |
| DQB1 | 77 | R | T | 0.357 | 0.365 | 0.98 | 0.88-1.08 | 6.35E-01 |  |
| DQB1 | 84 | E | Q | 0.363 | 0.420 | 0.78 | 0.70-0.87 | 3.02E-06 |  |
| DQB1 | 85 | V | L | 0.363 | 0.420 | 0.78 | 0.70-0.87 | 3.02E-06 |  |
| DQB1 | 86 | A | - | 0.338 | 0.373 | 0.85 | 0.76-0.95 | 2.69E-03 | 4.48E-08 |
| DQB1 | 86 | E | - | 0.637 | 0.580 | 1.28 | 1.15-1.42 | 3.02E-06 |
| DQB1 | 86 | G | - | 0.026 | 0.047 | 0.53 | 0.40-0.71 | 1.18E-05 |
| DQB1 | 87 | F | - | 0.178 | 0.199 | 0.87 | 0.77-0.99 | 3.51E-02 | 1.15E-05 |
| DQB1 | 87 | L | - | 0.637 | 0.580 | 1.28 | 1.15-1.42 | 3.02E-06 |
| DQB1 | 87 | Y | - | 0.185 | 0.221 | 0.79 | 0.70-0.90 | 3.47E-04 |
| DQB1 | 89 | G | T | 0.363 | 0.420 | 0.78 | 0.70-0.87 | 3.02E-06 |  |
| DQB1 | 90 | I | T | 0.363 | 0.420 | 0.78 | 0.70-0.87 | 3.02E-06 |  |
| DQB1 | 116 | I | V | 0.159 | 0.175 | 0.89 | 0.78-1.03 | 1.09E-01 |  |
| DQB1 | 125 | A | - | 0.637 | 0.580 | 1.28 | 1.15-1.42 | 3.02E-06 | 8.89E-06 |
| DQB1 | 125 | G | - | 0.204 | 0.246 | 0.78 | 0.69-0.88 | 6.92E-05 |
| DQB1 | 125 | S | - | 0.159 | 0.175 | 0.89 | 0.78-1.03 | 1.09E-01 |
| DQB1 | 126 | H | Q | 0.023 | 0.016 | 1.86 | 1.26-2.76 | 1.96E-03 |  |
| DQB1 | 130 | Q | R | 0.026 | 0.047 | 0.53 | 0.40-0.71 | 1.18E-05 |  |
| DQB1 | 135 | G | D | 0.101 | 0.084 | 1.23 | 1.03-1.46 | 2.23E-02 |  |
| DQB1 | 140 | T | A | 0.439 | 0.391 | 1.22 | 1.10-1.36 | 1.16E-04 |  |
| DQB1 | 167 | H | R | 0.194 | 0.213 | 0.91 | 0.80-1.04 | 1.67E-01 |  |
| DQB1 | 182 | N | S | 0.439 | 0.391 | 1.22 | 1.10-1.36 | 1.16E-04 |  |
| DQB1 | 185 | I | T | 0.252 | 0.183 | 1.47 | 1.30-1.67 | 8.88E-10 |  |
| DQB1 | 203 | V | I | 0.356 | 0.416 | 0.77 | 0.70-0.86 | 1.16E-06 |  |
| DQB1 | 220 | R | H | 0.363 | 0.420 | 0.78 | 0.70-0.87 | 3.02E-06 |  |
| DQB1 | 221 | Q | H | 0.363 | 0.420 | 0.78 | 0.70-0.87 | 3.02E-06 |  |
| DQB1 | 224 | R | Q | 0.159 | 0.175 | 0.89 | 0.78-1.03 | 1.09E-01 |  |
| DRB1 | -25 | R | K | 0.321 | 0.398 | 0.72 | 0.64-0.81 | 2.35E-08 |  |
| DRB1 | -24 | F | L | 0.159 | 0.156 | 0.98 | 0.84-1.14 | 7.85E-01 |  |
| DRB1 | -17 | T | A | 0.256 | 0.282 | 0.89 | 0.78-1.00 | 5.65E-02 |  |
| DRB1 | -16 | V | A | 0.321 | 0.398 | 0.72 | 0.64-0.81 | 2.35E-08 |  |
| DRB1 | -1 | S | A | 0.128 | 0.149 | 0.85 | 0.72-0.99 | 3.76E-02 |  |
| DRB1 | 4 | Q | R | 0.254 | 0.158 | 1.85 | 1.62-2.11 | 2.17E-19 |  |
| DRB1 | 9 | W | E | 0.492 | 0.416 | 1.39 | 1.25-1.55 | 1.15E-09 |  |
| DRB1 | 10 | Y | Q | 0.340 | 0.416 | 0.72 | 0.65-0.81 | 6.21E-09 |  |
| DRB1 | 11 | G | - | 0.246 | 0.149 | 1.90 | 1.66-2.18 | 1.98E-20 | 6.46E-20 |
| DRB1 | 11 | L | - | 0.103 | 0.114 | 0.89 | 0.75-1.05 | 1.65E-01 |
| DRB1 | 11 | P | - | 0.142 | 0.153 | 0.94 | 0.82-1.09 | 4.35E-01 |
| DRB1 | 11 | S | - | 0.340 | 0.416 | 0.72 | 0.65-0.81 | 6.21E-09 |
| DRB1 | 11 | V | - | 0.160 | 0.159 | 0.97 | 0.84-1.12 | 6.88E-01 |
| DRB1 | 12 | T | K | 0.340 | 0.416 | 0.72 | 0.65-0.81 | 6.21E-09 |  |
| DRB1 | 13 | F | - | 0.120 | 0.134 | 0.88 | 0.75-1.03 | 1.09E-01 | 3.72E-20 |
| DRB1 | 13 | G | - | 0.037 | 0.058 | 0.63 | 0.49-0.81 | 3.32E-04 |
| DRB1 | 13 | H | - | 0.152 | 0.148 | 0.98 | 0.85-1.13 | 7.72E-01 |
| DRB1 | 13 | R | - | 0.142 | 0.153 | 0.94 | 0.82-1.09 | 4.35E-01 |
| DRB1 | 13 | S | - | 0.303 | 0.359 | 0.78 | 0.69-0.87 | 1.17E-05 |
| DRB1 | 13 | Y | - | 0.246 | 0.149 | 1.90 | 1.66-2.18 | 1.98E-20 |
| DRB1 | 14 | K | E | 0.246 | 0.149 | 1.90 | 1.66-2.18 | 1.98E-20 |  |
| DRB1 | 16 | Y | H | 0.037 | 0.058 | 0.63 | 0.49-0.81 | 3.32E-04 |  |
| DRB1 | 25 | Q | R | 0.246 | 0.149 | 1.90 | 1.66-2.18 | 1.98E-20 |  |
| DRB1 | 26 | F | - | 0.762 | 0.721 | 1.24 | 1.10-1.39 | 3.84E-04 | 1.69E-03 |
| DRB1 | 26 | L | - | 0.124 | 0.147 | 0.82 | 0.70-0.95 | 9.64E-03 |
| DRB1 | 26 | Y | - | 0.115 | 0.132 | 0.84 | 0.72-0.99 | 3.62E-02 |
| DRB1 | 28 | E | D | 0.370 | 0.296 | 1.41 | 1.26-1.58 | 1.58E-09 |  |
| DRB1 | 30 | C | - | 0.103 | 0.114 | 0.89 | 0.75-1.05 | 1.65E-01 | 8.36E-20 |
| DRB1 | 30 | H | - | 0.012 | 0.022 | 0.50 | 0.32-0.76 | 1.50E-03 |
| DRB1 | 30 | L | - | 0.246 | 0.149 | 1.90 | 1.66-2.18 | 1.98E-20 |
| DRB1 | 30 | Y | - | 0.622 | 0.695 | 0.72 | 0.64-0.80 | 5.14E-09 |
| DRB1 | 31 | I | F | 0.111 | 0.124 | 0.88 | 0.75-1.04 | 1.28E-01 |  |
| DRB1 | 32 | H | Y | 0.244 | 0.299 | 0.74 | 0.65-0.83 | 4.78E-07 |  |
| DRB1 | 33 | H | N | 0.152 | 0.148 | 0.98 | 0.85-1.13 | 7.72E-01 |  |
| DRB1 | 37 | F | - | 0.276 | 0.179 | 1.78 | 1.56-2.02 | 1.46E-18 | 9.95E-19 |
| DRB1 | 37 | L | - | 0.012 | 0.022 | 0.50 | 0.32-0.76 | 1.50E-03 |
| DRB1 | 37 | N | - | 0.201 | 0.245 | 0.75 | 0.66-0.85 | 8.46E-06 |
| DRB1 | 37 | S | - | 0.246 | 0.267 | 0.90 | 0.80-1.02 | 9.86E-02 |
| DRB1 | 37 | Y | - | 0.265 | 0.287 | 0.90 | 0.80-1.02 | 8.59E-02 |
| DRB1 | 38 | L | V | 0.012 | 0.022 | 0.50 | 0.32-0.76 | 1.50E-03 |  |
| DRB1 | 47 | F | Y | 0.395 | 0.479 | 0.72 | 0.64-0.80 | 5.90E-10 |  |
| DRB1 | 57 | A | - | 0.030 | 0.030 | 0.98 | 0.72-1.34 | 9.06E-01 | 9.13E-14 |
| DRB1 | 57 | D | - | 0.663 | 0.739 | 0.69 | 0.61-0.77 | 2.48E-10 |
| DRB1 | 57 | S | - | 0.041 | 0.050 | 0.81 | 0.63-1.05 | 1.09E-01 |
| DRB1 | 57 | V | - | 0.266 | 0.180 | 1.67 | 1.47-1.90 | 5.04E-15 |
| DRB1 | 58 | E | A | 0.067 | 0.080 | 0.90 | 0.73-1.11 | 3.15E-01 |  |
| DRB1 | 60 | H | - | 0.030 | 0.030 | 0.98 | 0.72-1.34 | 9.06E-01 | 1.87E-14 |
| DRB1 | 60 | S | - | 0.266 | 0.180 | 1.67 | 1.47-1.90 | 5.04E-15 |
| DRB1 | 60 | Y | - | 0.704 | 0.790 | 0.62 | 0.55-0.71 | 7.48E-14 |
| DRB1 | 67 | F | - | 0.117 | 0.135 | 0.91 | 0.77-1.07 | 2.67E-01 | 2.98E-03 |
| DRB1 | 67 | I | - | 0.511 | 0.460 | 1.20 | 1.08-1.33 | 6.65E-04 |
| DRB1 | 67 | L | - | 0.373 | 0.405 | 0.86 | 0.78-0.96 | 6.01E-03 |
| DRB1 | 70 | D | - | 0.497 | 0.446 | 1.25 | 1.13-1.39 | 3.11E-05 | 1.54E-04 |
| DRB1 | 70 | Q | - | 0.456 | 0.504 | 0.81 | 0.73-0.9 | 8.58E-05 |
| DRB1 | 70 | R | - | 0.047 | 0.050 | 0.93 | 0.73-1.19 | 5.52E-01 |
| DRB1 | 71 | A | - | 0.123 | 0.141 | 0.86 | 0.74-1.00 | 5.27E-02 | 8.98E-05 |
| DRB1 | 71 | E | - | 0.119 | 0.138 | 0.79 | 0.68-0.93 | 3.82E-03 |
| DRB1 | 71 | K | - | 0.210 | 0.222 | 0.92 | 0.81-1.04 | 1.93E-01 |
| DRB1 | 71 | R | - | 0.549 | 0.499 | 1.27 | 1.14-1.41 | 1.18E-05 |
| DRB1 | 73 | G | A | 0.353 | 0.272 | 1.49 | 1.33-1.67 | 1.36E-11 |  |
| DRB1 | 74 | A | - | 0.575 | 0.646 | 0.73 | 0.66-0.82 | 1.81E-08 | 4.16E-19 |
| DRB1 | 74 | E | - | 0.047 | 0.047 | 0.99 | 0.77-1.27 | 9.24E-01 |
| DRB1 | 74 | L | - | 0.025 | 0.036 | 0.72 | 0.53-0.99 | 4.16E-02 |
| DRB1 | 74 | Q | - | 0.246 | 0.149 | 1.90 | 1.66-2.18 | 1.98E-20 |
| DRB1 | 74 | R | - | 0.107 | 0.123 | 0.85 | 0.72-1.00 | 4.97E-02 |
| DRB1 | 77 | N | T | 0.107 | 0.123 | 0.85 | 0.72-1.00 | 4.97E-02 |  |
| DRB1 | 78 | V | Y | 0.254 | 0.158 | 1.84 | 1.61-2.11 | 2.43E-19 |  |
| DRB1 | 85 | A | V | 0.030 | 0.034 | 0.82 | 0.61-1.11 | 1.94E-01 |  |
| DRB1 | 86 | V | G | 0.412 | 0.468 | 0.77 | 0.70-0.86 | 2.10E-06 |  |
| DRB1 | 96 | E | - | 0.103 | 0.114 | 0.89 | 0.75-1.05 | 1.56E-01 | 2.76E-01 |
| DRB1 | 96 | H | - | 0.594 | 0.575 | 1.10 | 0.99-1.22 | 7.89E-02 |
| DRB1 | 96 | Q | - | 0.151 | 0.163 | 0.94 | 0.81-1.08 | 3.85E-01 |
| DRB1 | 96 | Y | - | 0.152 | 0.148 | 0.98 | 0.85-1.13 | 7.81E-01 |
| DRB1 | 98 | E | K | 0.406 | 0.306 | 1.53 | 1.37-1.70 | 9.21E-14 |  |
| DRB1 | 104 | A | S | 0.406 | 0.306 | 1.53 | 1.37-1.70 | 9.21E-14 |  |
| DRB1 | 112 | Y | H | 0.030 | 0.030 | 0.99 | 0.72-1.35 | 9.44E-01 |  |
| DRB1 | 120 | N | S | 0.160 | 0.159 | 0.97 | 0.84-1.12 | 6.97E-01 |  |
| DRB1 | 133 | L | R | 0.142 | 0.153 | 0.94 | 0.82-1.09 | 4.41E-01 |  |
| DRB1 | 140 | A | T | 0.500 | 0.425 | 1.38 | 1.24-1.53 | 2.92E-09 |  |
| DRB1 | 142 | M | V | 0.142 | 0.153 | 0.94 | 0.82-1.09 | 4.41E-01 |  |
| DRB1 | 149 | H | Q | 0.340 | 0.416 | 0.72 | 0.65-0.81 | 5.98E-09 |  |
| DRB1 | 166 | Q | R | 0.005 | 0.010 | 0.40 | 0.18-0.90 | 2.68E-02 |  |
| DRB1 | 180 | L | V | 0.170 | 0.168 | 0.96 | 0.84-1.10 | 5.54E-01 |  |
| DRB1 | 181 | M | T | 0.253 | 0.166 | 1.75 | 1.54-1.99 | 5.51E-18 |  |
| DRB1 | 233 | R | T | 0.321 | 0.391 | 0.73 | 0.66-0.82 | 2.12E-08 |  |
