# Supplementary material for: Psoriasis Patients Are Enriched for Genetic Variants That Protect against HIV-1 Disease
Source: PLoS Genet. 2012 Feb 16;8(2):e1002514. doi: 10.1371/journal.pgen.1002514 (PMC3343879; doi:10.1371/journal.pgen.1002514)
Supplement: Table S5 — Amino acid residues identified by stepwise logistic regression as independently associated with psoriasis. (DOC) [file pgen.1002514.s005.doc]

Table S5. Amino acid residues identified by stepwise logistic regression as independently associated with psoriasis.

| Locus | Amino acid and position | Frequency in cases | Frequency in controls | Stepwise Univariate | | Multivariate | |
| --- | --- | --- | --- | --- | --- | --- | --- |
|  |  |  |  | P value | OR | P value | OR |
| HLA C | 156W | 0.367 | 0.209 | 1.02E-52 | 2.119 | 1.40E-08 | 1.43 |
| HLA B | 97V | 0.126 | 0.039 | 6.23E-21 | 2.635 | 5.70E-26 | 3.31 |
| HLA B | 145L | 0.063 | 0.025 | 4.67E-10 | 2.276 | 7.85E-11 | 2.50 |
| HLA B | 67C | 0.154 | 0.124 | 1.09E-06 | 1.43 | 8.21E-09 | 1.57 |
| HLA B | 99Y | 0.037 | 0.016 | 1.53E-07 | 2.369 | 1.51E-06 | 2.31 |
| HLA A | 107G | 0.313 | 0.271 | 7.99E-06 | 1.288 | 2.45E-06 | 1.31 |
| HLA C | 24A | 0.536 | 0.436 | 2.15E-05 | 1.242 | 2.15E-05 | 1.24 |
