# Supplementary material for: Psoriasis Patients Are Enriched for Genetic Variants That Protect against HIV-1 Disease
Source: PLoS Genet. 2012 Feb 16;8(2):e1002514. doi: 10.1371/journal.pgen.1002514 (PMC3343879; doi:10.1371/journal.pgen.1002514)
Supplement: Table S6 — Comparison of p-values and odds ratios (ORs) of the top HLA alleles, amino acid residues, and HLA-C 3′ UTR deletion SNP in the 3 psoriasis cohorts used for this study. I2 represents the proportion of variation that is due to heterogeneity. Roughly, I2 values of 25, 50, and 75 indicate low, moderate, and high heterogeneity. Most variants demonstrate low heterogeneity with a consistent direction of association across the 3 cohorts. (DOC) [file pgen.1002514.s006.doc]

**Table S6. Comparison of p-values and odds ratios (ORs) of the top HLA alleles, amino acid residues, and HLA-C 3’ UTR deletion SNP in the 3 psoriasis cohorts used for this study.** I^2^ represents the proportion of variation that is due to heterogeneity. Roughly, I^2^ values of 25, 50, and 75 indicate low, moderate, and high heterogeneity. Most variants demonstrate low heterogeneity with a consistent direction of association across the 3 cohorts.

|  | **Cohort 1**  (1348 cases, 1368 controls) | | **Cohort 2**  (210 cases, 502 controls) | | **Cohort 3**  (169 cases, 1711 controls) | | Heterogeneity |
| --- | --- | --- | --- | --- | --- | --- | --- |
| Variant | P | OR | P | OR | P | OR | I^2^ |
| HLA C*06:02 | 6.32E-52 | 3.73 | 3.87E-10 | 3.09 | 2.66E-19 | 3.65 | 0 |
| HLA B*57:01 | 8.50E-26 | 3.5 | 2.49E-07 | 3.65 | 8.46E-13 | 4.02 | 0 |
| HLA DQA1*02:01 | 4.84E-21 | 2.03 | 2.06E-04 | 1.80 | - | - | 0 |
| HLA DQB1*03:03 | 8.79E-19 | 2.70 | 5.35E-05 | 2.63 | - | - | 0 |
| HLA DRB1*07:01 | 3.72E-18 | 1.95 | 1.49E-03 | 1.70 | - | - | 0 |
| HLA B*13:02 | 1.72E-13 | 3.20 | 5.27E-04 | 3.16 | 9.74E-02 | 1.62 | 54.27 |
| HLA A*01:01 | 5.46E-06 | 1.40 | 5.33E-02 | 1.35 | 1.07E-02 | 1.44 | 0 |
| HLA C*04:01 | 8.60E-04 | 0.73 | 3.18E-02 | 0.59 | 1.35E-05 | 0.33 | 76.88 |
| HLA B*07:02 | 1.69E-04 | 0.72 | 1.28E-02 | 0.58 | 1.45E-01 | 0.74 | 0 |
| HL B*40:01 | 4.06E-06 | 0.55 | 5.21E-01 | 0.82 | 4.24E-01 | 0.77 | 0 |
| HLA B*35 | 1.23E-03 | 0.70 | 2.02E-01 | 0.70 | 1.47E-03 | 0.44 | 27.10 |
| HLA B 67C | 8.26E-02 | 1.16 | 1.46E-03 | 1.73 | 4.67E-05 | 1.77 | 77.49 |
| HLA B 67F | 1.05E-04 | 0.77 | 2.91E-01 | 0.84 | 1.91E-03 | 0.64 | 0 |
| HLA B 67M | 4.76E-24 | 3.03 | 1.12E-06 | 3.08 | 3.73E-10 | 3.17 | 0 |
| HLA B 67S | 6.86E-02 | 0.90 | 2.22E-03 | 0.67 | 3.03E-03 | 0.70 | 70.46 |
| HLA B 67Y | 1.12E-03 | 0.77 | 1.11E-01 | 0.74 | 6.17E-01 | 0.91 | 0 |
| HLA B 70K | 2.14E-01 | 1.19 | 3.82E-02 | 1.80 | 7.84E-02 | 1.61 | 13.7 |
| HLA B 70N | 8.30E-07 | 0.73 | 5.95E-03 | 0.68 | 3.42E-05 | 0.59 | 18.18 |
| HLA B 70Q | 1.31E-03 | 0.77 | 1.11E-01 | 0.74 | 6.17E-01 | 0.91 | 0 |
| HLA B 70S | 4.53E-24 | 3.03 | 1.12E-06 | 3.08 | 3.73E-10 | 3.17 | 0 |
| HLA B 97N | 1.93E-01 | 1.20 | 3.82E-02 | 1.80 | 7.84E-02 | 1.61 | 9.73 |
| HLA B 97R | 1.04E-06 | 0.76 | 5.73E-04 | 0.63 | 3.64E-04 | 0.65 | 18.66 |
| HLA B 97S | 1.59E-06 | 0.73 | 8.65E-03 | 0.67 | 1.30E-01 | 0.80 | 0 |
| HLA B 97T | 5.03E-05 | 1.42 | 5.87E-03 | 1.68 | 6.76E-01 | 1.07 | 42.52 |
| HLA B 97V | 8.50E-26 | 3.50 | 2.49E-07 | 3.65 | 8.72E-14 | 4.26 | 0 |
| HLA B 97W | 4.00E-01 | 0.87 | 3.05E-01 | 1.36 | 2.61E-01 | 1.32 | 31.16 |
| HLA B 116Y | 1.615E-012 | 0.66 | 4.93E-03 | 0.68 | 1.30E-03 | 0.66 | 0 |
| HLA C 97W | 1.06E-31 | 2.12 | 1.61E-07 | 2.09 | 2.71E-10 | 1.71 | 54.42 |
| HLA C 156D | 7.41E-01 | 0.93 | 3.20E-01 | 0.50 | 3.07E-01 | 0.59 | 0 |
| HLA C 156L | 9.20E-14 | 0.65 | 1.05E-03 | 0.65 | 5.19E-03 | 0.78 | 30.53 |
| HLA C 156Q | 3.60E-02 | 0.72 | 1.03E-02 | 0.28 | 3.34E-01 | 0.78 | 44.52 |
| HLA C 156R | 4.71E-05 | 0.77 | 2.93E-01 | 0.86 | 3.67E-02 | 0.82 | 0 |
| HLA C 156W | 1.87E-38 | 2.43 | 1.29E-09 | 2.49 | 6.95E-10 | 1.70 | 82.62 |
| rs67384697 del | 3.41E-20 | 1.71 | 2.34E-04 | 1.59 | 4.29E-09 | 2.01 | 0.85 |
