# Supplementary material for: Psoriasis Patients Are Enriched for Genetic Variants That Protect against HIV-1 Disease
Source: PLoS Genet. 2012 Feb 16;8(2):e1002514. doi: 10.1371/journal.pgen.1002514 (PMC3343879; doi:10.1371/journal.pgen.1002514)
Supplement: Table S7 — Stepwise conditioning of HLA-C*06:02 association signal on the coding amino acids of HLA-C*06:02. The significant residual association indicates that the association of HLA-C*06:02 with psoriasis cannot be entirely explained by coding differences between HLA-C*06:02 and other HLA-C alleles. Therefore, the association of HLA-C*06:02 with psoriasis likely reflects the contribution of other variants in LD with HLA-C*06:02 such as HLA-B*57:01. (DOC) [file pgen.1002514.s007.doc]

**Table S7. Stepwise conditioning of HLA-C*06:02 association signal on the coding amino acids of HLA-C*06:02.** The significant residual association indicates that the association of HLA-C*06:02 with psoriasis cannot be entirely explained by coding differences between HLA-C*06:02 and other HLA-C alleles. Therefore, the association of HLA-C*06:02 with psoriasis likely reflects the contribution of other variants in LD with HLA-C*06:02 such as HLA-B*57:01.

| Stepwise condition on amino acid of HLA-C | P-value for HLA-C*06:02 | OR | 95% CI |
| --- | --- | --- | --- |
|  | 2.91E-77 | 3.57 | 3.12-4.08 |
| C156W | 2.34E-37 | 2.80 | 2.39-3.28 |
| C24S | 7.30E-30 | 2.62 | 2.22-3.10 |
| C-14L | 6.92E-22 | 2.41 | 2.02-2.89 |
